# Treatment response in rheumatoid arthritis is predicted by the microbiome: a large observational study in UK DMARD-naive patients

**DOI:** 10.1093/rheumatology/keae045

**Published:** 2024-01-30

**Authors:** Nathan P Danckert, Maxim B Freidin, Isabelle Granville Smith, Philippa M Wells, Maryam Kazemi Naeini, Alessia Visconti, Roger Compte, Alexander MacGregor, Frances M K Williams

**Affiliations:** Department of Twin Research and Genetic Epidemiology, School of Life Course & Population Sciences, King’s College London, London, UK; Department of Biology, School of Biological and Behavioural Sciences, Queen Mary University of London, London, UK; Department of Twin Research and Genetic Epidemiology, School of Life Course & Population Sciences, King’s College London, London, UK; UK Dementia Research Institute, Imperial College London, London, UK; Department of Brain Sciences, Imperial College London, London, UK; Department of Twin Research and Genetic Epidemiology, School of Life Course & Population Sciences, King’s College London, London, UK; Department of Twin Research and Genetic Epidemiology, School of Life Course & Population Sciences, King’s College London, London, UK; Department of Twin Research and Genetic Epidemiology, School of Life Course & Population Sciences, King’s College London, London, UK; Norwich Medical School, University of East Anglia, Norwich, UK; Rheumatology Department, Norfolk and Norwich University Hospitals NHS Trust, Norwich, UK; Department of Twin Research and Genetic Epidemiology, School of Life Course & Population Sciences, King’s College London, London, UK; Guy’s and St Thomas’ NHS Trust, London, UK

**Keywords:** gut microbiome, rheumatoid arthritis, disease-modifying antirheumatic drugs, shotgun metagenomic sequencing, minimal clinically important improvement

## Abstract

**Objectives:**

Disease-modifying antirheumatic drugs (DMARDs) are a first-line treatment in rheumatoid arthritis (RA). Treatment response to DMARDs is patient-specific, dose efficacy is difficult to predict and long-term results are variable. The gut microbiota are known to play a pivotal role in prodromal and early-disease RA, manifested by *Prevotella* spp. enrichment. The clinical response to therapy may be mediated by microbiota, and large-scale studies assessing the microbiome are few. This study assessed whether microbiome signals were associated with, and predictive of, patient response to DMARD treatment. Accurate early identification of those who will respond poorly to DMARD therapy would allow selection of alternative treatment (e.g. biologic therapy) and potentially improve patient outcome.

**Methods:**

A multicentre, longitudinal, observational study of stool- and saliva microbiome was performed in DMARD-naive, newly diagnosed RA patients during introduction of DMARD treatment. Clinical data and samples were collected at baseline (*n* = 144) in DMARD-naive patients and at six weeks (*n* = 117) and 12 weeks (*n* = 95) into DMARD therapy. Samples collected (*n* = 365 stool, *n* = 365 saliva) underwent shotgun sequencing. Disease activity measures were collected at each timepoint and minimal clinically important improvement determined.

**Results:**

In total, 26 stool microbes were found to decrease in those manifesting a minimal clinically important improvement. *Prevotella* spp. and *Streptococcus* spp. were the predominant taxa to decline following six weeks and 12 weeks of DMARDs, respectively. Furthermore, baseline microbiota of DMARD-naive patients were indicative of future response.

**Conclusion:**

DMARDs appear to restore a perturbed microbiome to a eubiotic state. Moreover, microbiome status can be used to predict likelihood of patient response to DMARD.

Rheumatology key messages

- RA microbiota differentiates response to DMARDs.
- *Prevotella* spp. reduced in responders following six weeks treatment.
- *Streptococcus* spp. reduced in responders following 12 weeks.
- DMARDs appear to restore a perturbed microbiome to a more eubiotic state.

## Introduction

Rheumatoid arthritis (RA) is an autoimmune, multi-system inflammatory disorder usually treated with disease-modifying antirheumatic drugs (DMARDs) and biologic therapy. Known to be substantially heritable [1], aetiology is a complex interaction of genetic and environmental triggers, not wholly defined. The autoimmune pathogenesis of RA takes place over many years prior to symptom onset [2]. The importance of the microbiota in the development and maintenance immune system function is well recognised [3]. Aberrant immune responsiveness may result from an anormal microbiome and there is intense research activity to determine whether gut and oral microbes play a role in RA pathogenesis [4].

We previously used an RA polygenic risk score (PRS) in non-affected individuals to show host genetic makeup associates with enrichment for *Prevotella* spp. in the stool of TwinsUK participants who had higher PRS for RA [5]. PRS minimizes confounding by RA and treatment in association studies. Other groups have shown RA patients manifest high levels of *Prevotella copri (P. copri)* in stool early in symptom onset [6, 7]. Furthermore, serum anti-*P. copri* antibodies and *P. copri*-derived proteins were detectable in joint synovial fluid of RA patients [8].

DMARDs are currently the first choice of treatment for patients diagnosed with RA in the UK [9]. In some patients, disease activity is reduced, joint damage viewed radiographically is slowed, and remission may be achieved [10]. Methotrexate is the most prescribed DMARD; it inhibits multiple inflammatory pathways that are active in RA [9]. Orally administered methotrexate is metabolised by the gut microbiome and individuals’ variability of microbiome abundance and diversity likely contributes to differences in medication efficacy [9]. Treatment strategies in RA currently involve introducing DMARDs at a low dose with escalation over months. Methotrexate monotherapy results in less than half of patients responding well to their prescription [11]. In patients non-responsive to first-line therapy, the iterative process of drug assaying means time is lost taking DMARD medication to which some patients never respond well, irrespective of dose. As early and aggressive therapy (i.e. treat-to-target) improves long-term outcomes, advances in patient stratification towards therapies from which they will benefit are welcome.

Predicting patient response to DMARD therapy via the microbiome would be invaluable for prognosis. Three metagenome studies have reported microbiota predicted DMARD response [12–14], although further validation is required before clinical application. In the present study, we sought to determine whether the microbiome (oral and gut) before treatment in RA predicted response to DMARDs. In this longitudinal, observational, multicentre study we recruited newly diagnosed RA patients and obtained oral and gut samples at DMARD-naive baseline and at follow-up six weeks and 12 weeks later. We examined metagenomes to determine whether microbiome composition and functionality of DMARD-naive patients early during RA were predictive of responses to treatment. Furthermore, we compared the microbiota composition of newly diagnosed RA patients with long-term DMARD-treated RA participants from the Norfolk Arthritis Register (NOAR).

## Materials and methods

### Study design and participants

The Influence of Methotrexate on the Rheumatoid Arthritis Microbiome (IMRABIOME) study is a longitudinal, observational study of microbiota observed in DMARD-naive newly diagnosed RA patients who had inflammatory arthritis symptoms for <12 months and fulfilled the 2010 European League Against Rheumatism (EULAR)/American College of Rheumatology (ACR) classification criteria for RA [15]. Recruitment was undertaken between April 2017 and July 2019 from 12 outpatient rheumatology clinics in England, UK. All participants were DMARD-naive at recruitment but due to commence DMARD treatment, prescribed by their consultant rheumatologist in accordance with UK standard treatment and National Institute for Health and Care Excellence (NICE) guidelines. Inclusion criteria comprised: age of 18 or over, with new-onset RA and having received rheumatologist advice to commence DMARD therapy. All interventions and medications were recorded in the study database including corticosteroid treatment (prednisolone <20 mg per day) and non-steroidal anti-inflammatory drugs (NSAIDs). Concurrent non-rheumatic disorders and their treatment were recorded. Exclusion criteria included previous exposure to DMARD or biologic therapy; corticosteroid treatment equivalent to prednisolone ≥20 mg/day, significant comorbidities (e.g. severe congestive heart failure; renal, hepatic or malignant disease); pregnancy; and enrolment in a clinical trial.

DMARD-naive participants were assessed at baseline and at six and 12 weeks after commencing DMARD therapy. Intestinal (stool) and saliva samples clinical markers and relevant clinical, demographic and lifestyle information were collected. Participants completed a variety of health, well-being and activities of daily functioning questionnaires. Written informed consent was obtained from all participants and the Declaration of Helsinki (1996) was followed. Study approval was granted by the Health Research Authority (HRA) Research Ethics Committee (REC) IRAS, project 212604. Sample collection is detailed in the Supplementary Methods, available at *Rheumatology* online.

### Microbiome profiling and collection

Shotgun metagenomic analysis was performed on stool and saliva samples collected from study participants, as described in the Supplementary Methods, available at *Rheumatology* online. In short, samples were sent to TwinsUK laboratories within 24 h of collection (stool) and 2 h of collection (saliva), where they were stored at −80°C until processing. Genomic DNA was extracted and processed at Clinical Microbiomics (Copenhagen, Denmark) for shotgun metagenomics using 2 × 150 bp paired-end chemistry on an Illumina NovaSeq 6000 platform. Quality control of sequencing data was performed by YAMP [16]. Taxonomic profiling was carried out using kracken2/bracken [17], and HUMAnN3 [18] was used for functional profiling (UniRef90 reference database).

### Microbiome profiling: NOAR cohort

Patients with chronic RA were obtained from the Norfolk Arthritis Register (NOAR) (Participant Characteristics Supplementary Table 1, available at *Rheumatology* online). NOAR samples (212 stool and saliva samples) were collected from participants on the registry with RA who had been taking DMARD therapy >12 months [19], with all participants completing the clinical questionnaire. Samples were sent to Clinical Microbiomics (Denmark) and processed through default settings in YAMP, as outlined above.

### Statistical analysis

#### Alpha- and beta-diversity

Chao1 richness, Shannon- and Simpson diversity index were calculated in the Phyloseq R package (v1.40.0) [20]. Alpha-diversity metrics were compared at each time point using linear mixed modelling, adjusting for clinical and demographic covariates such as age, sex, body mass index (BMI), smoking status, ethnicity and hospital centre, within the Vegan R package (v2.6–4) [21].

Before beta-diversity, data were filtered using PERFect (v1.10.0) [22], a novel permutation filter approach based on statistical hypothesis testing in taxa reduction. PERFect removes potential sequencing contaminant and taxa with minimal impact on the overall covariation within microbiome datasets.

Data were normalized with an arcsine square root transformation and clusters were visualized using Bray–Curtis dissimilarity and principal coordinates analysis (PCoA) using Vegan. Permutational Multivariate Analysis of Variation (PERMANOVA) was used to statistically compare cluster centroids and repeat measures were considered using *strata* parameter, where applicable.

#### Differential abundance

Comparison of different taxonomic abundances between time points and clinical data was carried out using the limma: voom pipeline (v3.52.4) [23, 24] after normalizing abundances using GMPR (v0.1.3) [25]. Statistical significance threshold was set at 10% false discovery rate (FDR). Clinical Disease Activity Index (CDAI) score was used to classify patients into two groups according to minimal clinically important improvement (MCII), as defined in Gupta and colleagues [13], to determine preditive DMARD treatment response in baseline microbiota. This allowed the comparison of those with a meaningful clinical response to DMARD therapy with participants that did not (Supplementary Methods, available at *Rheumatology* online).

#### Predictive model for MCII

A sparse partial least squares discriminant analysis [(s)PLS-DA] model was developed to predict MCII in microbiome data, using the MixOmics package (v6.20.0) [26]. Baseline abundance and MCII status established after six weeks of DMARD treatment were used to train and test the model. Samples were randomly split into two equal subsets, between those that did and did not achieve MCII (*n* = 53, with *n* = 32 MCII+ and *n* = 21 MCII-), and confusion matrices and area under the curve (AUC) plots were used to evaluate model performance. An additional microbiome dataset obtained from Gupta and colleagues [13] was used as a validation RA cohort for the (s)PLS-DA model.

## Results

### Sample description

The IMRABIOME cohort comprised 144 RA participants assessed at baseline (*n* = 144), at six weeks (*n* = 117) and at 12 weeks (*n* = 95) follow-up. A total of 72.9% of participants were female and 63.8% northern Europeans. Stool and saliva samples were collected at all timepoints for metagenomics alongside clinical markers, demographic and lifestyle information. All data were available at all three timepoints for 92 participants, 27 had data available at two time points (i.e. baseline and six weeks, or 12 weeks); and 26 with data available only at a single time point (i.e. only baseline).

Demographics and clinical characteristics are presented in Table 1.

**Table 1.** Clinical and demographic characteristics of RA patients

Trait		Baseline, *n* = 144	6 weeks, *n* = 117	12 weeks, *n* = 95
Sex^a^	Female, *n* (%)	105 (72.4)		
	Male, *n* (%)	38 (26.2)		
	Unknown, *n* (%)	2 (1.4)		
Age, mean±SD		53.8 ± 13.9		
Smoking status^a^	Never, *n* (%)	66 (45.5)		
	Ever, *n* (%)	52 (35.9)		
	Current, *n* (%)	24 (16.6)		
	Unknown, *n* (%)	3 (2.1)		
Diet	Diet including meat and fish, *n* (%)	65 (49.3)		
	Diet excluding meat and fish, *n* (%)	67 (50.7)		
Body mass index, mean±SD		28.5 ± 6.1		
Ethnicity	Northern European, *n* (%)	92 (63.8)		
	South Asian, *n* (%)	17 (11.8)		
	East Asian, *n* (%)	5 (3.4)		
	Black African, *n* (%)	9 (6.2)		
	Black Caribbean, *n* (%)	7 (4.8)		
	Mixed – Other, *n* (%)	4 (2.7)		
	Unknown, *n* (%)	10 (6.9)		
Hospital Centre	North, *n* (%)	13 (9.0)		
	South, *n* (%)	91 (63.1)		
	East, *n* (%)	19 (13.1)		
	West, *n* (%)	21 (14.5)		
Anti-CCP	Positive, *n* (%)	88 (65.7)		
	Negative, *n* (%)	46 (34.3)		
Tender joint score	Median (min, max)	7 (0; 28)	3 (0; 27)	2 (0; 28)
Swollen joint score	Median (min, max)	5 (0; 28)	1 (0; 22)	1 (0; 23)
C-reactive protein	Median (min, max)	7.1 (1; 209)	4 (1; 78)	4 (1; 122)
DAS28 score	Median (min, max)	4.9 (1.4; 8.2)	3.5 (0.0; 7.3)	3.8 (0.7; 7.7)
	High, *n* (%)	65 (45.1)	22 (18.8)	19 (20.0)
	Moderate, *n* (%)	54 (37.5)	42 (35.9)	21 (22.1)
	Low, *n* (%)	22 (15.3)	50 (42.7)	51 (53.7)
	Unknown, *n* (%)	3 (2.1)	3 (2.6)	4 (4.2)
	Disease activity decreased *vs* baseline^b^		54/112 (48.2)	44/88 (50.0)
CDAI score	Median (min, max)	25 (0; 73)	13 (0; 65)	8.5 (0; 61)
	High, *n* (%)	80 (55.6)	30 (25.6)	21 (22.1)
	Moderate, *n* (%)	33 (22.9)	34 (29.1)	17 (17.9)
	Low, *n* (%)	23 (16.0)	32 (27.4)	26 (27.4)
	Remission/no active disease, *n* (%)	4 (2.8)	17 (14.5)	26 (27.4)
	Unknown, *n* (%)	4 (2.8)	4 (3.4)	5 (5.3)
	Disease activity decreased *vs* baseline^c^		64/106 (60.4)	49/82 (59.8)
DMARD Treatment				
Monotherapy	Methotrexate – monotherapy, *n*	75	54	32
	Sulfasalazine – monotherapy, *n*	4	3	3
	Hydroxychloroquine – monotherapy, *n*	4	4	3
Combination	Methotrexate and Sulfasalazine, *n*	7	12	6
	Methotrexate and Hydroxychloroquine, *n*	51	37	37
	Sulfasalazine and Hydroxychloroquine, *n*	2	2	3
	Methotrexate, Sulfasalazine and Hydroxychloroquine, *n*	1	2	2
**Total use (any)**	Methotrexate, *n* (%)	134/144 (93.1)	105/113 (92.9)	77/86 (89.5)
	Sulfasalazine, *n* (%)	16/144 (11.1)	19/109 (17.4)	14/85 (16.5)
	Hydroxychloroquine, *n* (%)	58/144 (40.3)	45/110 (40.9)	45/87 (51.7)

a One patient did not have microbiome data at baseline, and at follow-up only; accordingly, proportions for sex and smoking at baseline are estimated for 145 individuals.

b Patients’ disease activity category decreased from high to moderate or low, from moderate to low.

c CDAI score decreased for at least 1 unit for those who had low activity, at least 6 units for those who had moderate activity, and at least 12 units for those who had high activity.

RA: rheumatoid arthritis; BMI: body mass index; DAS: Disease Activity Score.

### Alpha-diversity

Patient demographics impact on alpha-diversity was examined using Chao1 richness, Shannon- and Simpson’s diversity index, in stool and saliva. No significant difference was seen in alpha-diversity metrics at any timepoint for either sample type (*P* >0.05). Smoking status was significant negatively associated with Shannon and Simpson indices across all three timepoints in saliva (Supplementary Table 2, available at *Rheumatology* online), with current and previous smokers having a reduced diversity compared with non-smokers. Smoking status did not significantly associate with alpha-diversity in the stool microbiome.

Disease activity measured by Disease Activity Score (DAS)28 and CDAI was negatively associated with all three alpha-diversity metrics in stool (Table 2). Sulfasalazine reduced richness in the gut at six weeks (*P* = 0.015), no further DMARD influence was seen in gut or saliva alpha-diversity. Other demographics, such as sex, associated with Shannon diversity at baseline and six weeks, with males showing a reduced diversity compared with females (*P* < 0.05).

**Table 2.** Disease activity and microbiome alpha-diversity

Alpha diversity measure	Disease activity and severity scores	Baseline	6 weeks	12 weeks
Chao1 richness	DAS28	β = −161.64 ± 42.55; *P* = 0.0002	β = −142.15 ± 38.97; *P* = 0.0004	β = −197.98 ± 55.06; *P* = 0.0005
	CDAI	β = −16.03 ± 4.12; *P* = 0.0001	β = −15.38 ± 4.48; *P* = 0.0008	β = −19.92 ± 6.10; *P* = 0.001
Shannon index	DAS28	β = −0.59 ± 0.19; *P* = 0.003	β = −0.46 ± 0.21; *P* = 0.027	β = −0.49 ± 0.29; *P* = 0.094
	CDAI	β = −5.51 ± 2.07; *P* = 0.009	β = −3.46 ± 1.83; *P* = 0.062	β = −5.14 ± 2.69; *P* = 0.059
Simpson index	DAS28	β = −2.20 ± 1.11; *P* = 0.050	β = −3.19 ± 1.11; *P* = 0.005	β = −2.01 ± 1.59; *P* = 0.211
	CDAI	β = −19.10 ± 12.03; *P* = 0.114	β = −29.56 ± 9.76; *P* = 0.003	β = −24.75 ± 14.11; *P* = 0.095

Alpha diversity was calculated from stool metagenomes. Estimates ± standard error and *P*-values for multiple regression of RA activity scores on Chao1, Shannon and Simpson indices; adjusted for age, sex, BMI and smoking status.

BMI: body mass index; CDAI: Clinical Disease Activity Index; DAS: Disease Activity Score; RA: rheumatoid arthritis.

### Beta-diversity

Microbial data were analysed with arcsine transformed PERFect-filtered data using PCoA to identify outliers before statistical comparisons. After removing outliers, we recalculated Bray–Curtis dissimilarity and used PERMANOVA models to establish demographic and clinical factors contributing to gut and saliva microbiome variance. Using univariate and multivariate models we assessed impacts of age, sex, BMI, smoking, hospital centre, ethnicity, disease severity as measured by DAS28 score, and DMARD use, at six and 12 weeks.

In multivariate models, the saliva microbiome was significantly different in participant smoking status at all timepoints (*P* < 0.001, Supplementary Table 3, available at *Rheumatology* online). Age was the only other variable to significantly differ in the saliva microbiome, which occurred at baseline (*P* = 0.016). In the gut, age and ethnicity significantly differed at all timepoints, while BMI and DAS28 also differed at individual sampling points (Table 3). DMARD monotherapy did not significantly impact microbial communities in stool or saliva models; however, at six weeks’ follow-up, for participants on combination therapy, the methotrexate component had a significant effect on gut microbiome beta-diversity.

**Table 3.** Disease activity, patient characteristics and gut microbiome variance

Trait	Model	Baseline		Six weeks		12 weeks	
		R^2^	*P*-value	R^2^	*P*-value	R^2^	*P*-value
Age	Univariate	1.4	0.0186	1.8	0.0052	2.5	0.0031
	Adjusted	1.3	0.0288	2.0	0.0091	3.6	0.0002
Sex	Univariate	1.3	0.0300	1.6	0.0205	1.6	0.0617
	Adjusted	1.0	0.1091	1.8	0.0222	1.5	0.1591
BMI	Univariate	1.6	0.0079	0.9	0.2834	1.0	0.5027
	Adjusted	1.4	0.0193	1.1	0.2833	0.9	0.6611
Smoking	Univariate	1.0	0.8588	1.4	0.8074	1.7	0.7808
	Adjusted	1.2	0.7127	1.6	0.7723	2.4	0.4017
Diet	Univariate	0.3	0.9529	—	—	—	—
	Adjusted	0.4	0.9738	—	—	—	—
Site (hospital)	Univariate	2.2	0.3239	3.1	0.1496	4.1	0.0810
	Adjusted	2.3	0.3476	3.1	0.3599	4.9	0.0505
Ethnicity	Univariate	7.1	0.0003	7.6	0.0011	8.8	0.0033
	Adjusted	6.8	0.0002	7.3	0.0083	8.4	0.0262
DAS28 score	Univariate	1.4	0.0173	1.6	0.0257	3.4	0.0004
	Adjusted	0.9	0.1824	0.8	0.5704	2.3	0.0154
Tender joint score	Univariate	0.9	0.1605	—	—	—	—
	Adjusted	1.1	0.0649	—	—	—	—
Swollen joint score	Univariate	0.5	0.6861	—	—	—	—
	Adjusted	1.1	0.0728	—	—	—	—
C-reactive protein	Univariate	0.5	0.5949	—	—	—	—
	Adjusted	0.6	0.5206	—	—	—	—
Methotrexate–mono	Univariate	—	—	3.4	0.0139	3.2	0.2030
	Adjusted	—	—	2.2	0.2237	2.4	0.4633
Sulfasalazine–mono	Univariate	—	—	1.0	0.2898	1.2	0.4938
	Adjusted	—	—	—	—	—	—
Hydroxychloroquine– mono	Univariate	—	—	1.2	0.8384	2.1	0.5215
	Adjusted	—	—	—	—	—	—
Use of methotrexate	Univariate	—	—	1.6	0.0287	3.4	0.3447
	Adjusted	—	—	1.8	0.0353	1.4	0.2626
Use of sulfasalazine	Univariate	—	—	1.6	0.0283	2.1	0.0211
	Adjusted	—	—	1.1	0.2645	1.5	0.1735
Use of hydroxychloroquine	Univariate	—	—	1.5	0.0452	1.1	0.3945
	Adjusted	—	—	0.8	0.6492	1.2	0.3827

Bray–Curtis dissimilarity was calculated using stool microbiome at species level; because of high correlation between DAS28 and CDAI scores, we chose to analyse DAS28 score only.

BMI: body mass index; CDAI: Clinical Disease Activity Index; DAS28: Disease Activity Score 28; mono: monotherapy.

### Differential abundance

Differential abundance (DA) was carried out using the limma: voom approach with abundance normalized with the GMPR, characterized by improved power and control of false positives compared with other normalization techniques. First, we checked for microbiome abundance changes across time points using linear contrast for pairwise comparisons (Supplementary Tables 4 and 5, available at *Rheumatology* online). Differences in the saliva microbiome were inconsistent, as such we focused on the gut microbiome (saliva data in Supplementary Tables 6 and 7, available at *Rheumatology* online). In the gut, when comparing timepoints, we found more differential abundant taxa between baseline and 12 weeks, than baseline and six weeks (Supplementary Table 8, available at *Rheumatology* online). No significant findings were discovered while comparing follow-up time points. Next, we compared our study cohort of early-RA patients to NOAR, a long-term treatment cohort. DMARD-naive baseline, several weeks of DMARD treatment (i.e. combination of six-week and 12-week samples) and long-term (>1 year) use of DMARD (NOAR sample) were compared (FDR < 0.10) (Supplementary Tables 9–11, available at *Rheumatology* online). In total, 32 taxa differed in abundance between long-term DMARD use and several weeks of DMARD use. In contrast, 86 taxa significantly decreased in long-term DMARD treated participants compared with DMARD-naive baseline, with *Porphyromonas gingivalis* and 15 *Prevotella* spp. among the reduced taxa.

Minimal clinically important improvement (MCII) was used to assess microbiome change based on patient response to DMARD treatment. At baseline, there were no detectable microbiome differences between those that achieved MCII and those who did not. After six weeks of DMARD treatment, 18 taxa differed between MCII groups, of which 13 decreased and five increased in abundance, in those that achieved MCII *vs* those that did not. Of the 13 decreased taxa, seven (54%) were *Prevotella* spp. At 12 weeks treatment, a further 13 taxa significantly decreased when comparing participants that fulfilled MCII compared with those that did not, of which 10 were *Streptococcus* spp. (Table 5). Overall, these findings led us to conclude that the gut microbiome normalises in RA patients with longer treatment, but only in patients who respond to DMARD treatment.

**Table 5.** Patient response (MCII+/MCII-) and differentially abundant taxa following 12 weeks of DMARD treatment

Taxa	MCII+ vs MCII- @ 12 weeks		
	logFC	*P*-value	adj. *P*-value
*Gemella haemolysans*	−1.7	0.000	0.094
*Staphylococcus epidermidis*	−1.45	0.000	0.094
*Veillonella parvula*	−1.92	0.000	0.094
*Streptococcus mitis*	−1.76	0.000	0.094
*Streptococcus* sp. oral taxon 061	−1.75	0.001	0.094
*Streptococcus gordonii*	−1.61	0.001	0.094
*Streptococcus oralis*	−1.59	0.001	0.094
*Streptococcus* sp. A12	−1.85	0.001	0.094
*Streptococcus lactarius*	−1.78	0.001	0.094
*Streptococcus gallolyticus*	−1.52	0.001	0.094
*Streptococcus pneumoniae*	−1.21	0.001	0.094
*Streptococcus parasanguinis*	−1.89	0.001	0.094
*Streptococcus* sp. LPB0220	−2.02	0.001	0.094

Differentially abundant taxa between RA patients who achieved minimum clinically important improvement (MCII+) and who did not (MCII-) following 12 weeks of DMARD treatment. Linear contrasts have been constructed and tested for significance using voom: limma approach combined with GMPR normalisation. Adjustment for multiple testing was done using Benjamini–Hochberg false discovery rate within each taxonomic level. Reported are findings with FDR <10% in at least one contrast. No significant results were obtained for comparisons between MCII+ and MCII- at baseline.

### Anti-CCP

Anti-cyclic citrullinated peptide (anti-CCP) values at baseline were positive for 88 participants and negative for 46 participants (defined as <20 u/ml antibody negative, ≥20 u/ml positive), Table 1. We explored the relationship between anti-CCP antibodies and smoking in saliva microbiota using linear regression models. The inclusion of anti-CCP did not significantly alter Shannon diversity (*P* = 0.11) or Chao1 richness (*P* = 0.67) measures. Furthermore, univariate (*P* = 0.22) and multivariate (*P* = 0.20) PERMANOVA statistics suggested anti-CCP did not significantly impact the saliva microbiota diversity. The inclusion of anti-CCP in the gut microbiota linear regression did not improve model outcomes, with no significant association between anti-CCP and the gut microbiota (Shannon *P* = 0.15, Chao1 *P* = 0.07). Prevotella species were, however, elevated in individuals positive for anti-CCP antibodies (Supplementary Table 12, available at *Rheumatology* online).

### Predictive model for MCII

**Figure 1.**
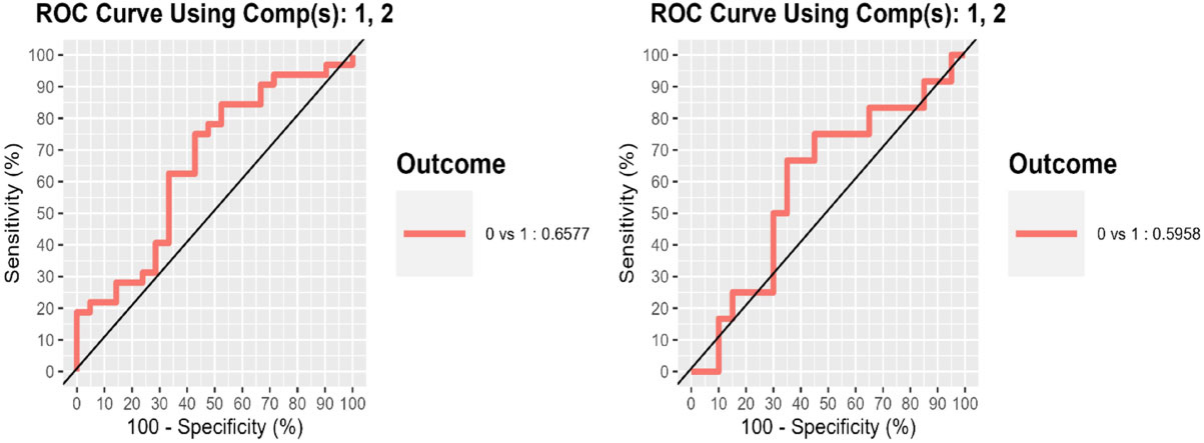
Area under the ROC curve for predictive model of MCII after short-term treatment. Left panel is for the test sample resulted from the split of our dataset into random train and test samples. Right panel is for external data [13]

We applied sPLSDA analysis to develop a model to predict MCII status upon short-term treatment (see ‘Materials and methods’). We used baseline species abundances and MCII status as established at six weeks. Furthermore, using Kegg Orthology and Metacyc database microbial gene and biochemical pathways, predictive potential was also explored. To generate train and test samples, we randomly split the dataset with available information into two equal subsets, *n* = 53 each, with *n* = 32 who achieved MCII and *n* = 21 who did not. In the gut, the final model for microbes provided AUC = 0.66 (*P* = 0.0539); while for Gupta and colleagues [13], AUC = 0.60 (*P* = 0.3706) (Fig. 1). Saliva samples manifested an AUC = 0.61 (*P* = 0.14) for microbes and AUC = 0.69 (*P* = 0.033) for Metacyc pathways, while KO genes for stool and saliva had AUCs <0.55 (Supplementary Tables 13–15, available at *Rheumatology* online).

## Discussion

The microbiome has an established and prominent role in RA disease and early-onset development [27, 28]. Our research focused on early-disease RA patients who were DMARD-naive and examined microbiome changes during initiation of DMARD therapy in a large-scale observation study of oral and gut microbiome in 144 individuals. DMARD therapy is recommended early in disease to improve patient outcomes, but currently a trial-and-error approach is used, with drug dose escalation if response is poor. The ability to determine who will respond to DMARDs would enable a personalised approach to treatment selection, rather than the ubiquitous administration of methotrexate in new-onset RA in the UK. Our interest was in being able to predict how patients will respond to DMARD therapy. There were two main findings from our study. First, microbial abundance among patients that attained MCII (MCII+) and those that did not (MCII-), were different at 6 and 12 weeks of DMARD treatment. In total, 26 different stool microbes decreased in MCII+ compared with MCII-. *Prevotella* spp. and *Streptococcus* spp. were the predominant taxa to decline after six weeks and 12 weeks, respectively. Interestingly, diet preference had limited impact on the stool microbiota and abundance differences seen in this study, with no changes to microbiota seen (Tables 4 and 5). Second, we found the DMARD-naive microbiome was indicative of clinical response. Metacyc pathways best predicted MCII+ from DMARD-naive samples, particularly saliva, providing a promising target for metabolomics. Furthermore, we found 86 microbes decreased significantly in long-term DMARD-exposed participants from the NOAR cohort, when compared with DMARD-naive patients; *Prevotella* spp. accounted for ∼20% of species in decline.

**Table 4.** Patient response (MCII+/MCII-) and differentially abundant taxa during the first six weeks of DMARD treatment

Taxa	MCII+ vs MCII- @ Six weeks		
	logFC	*P*-value	adj. *P*-value
*Phocaeicola salanitronis*	−1.73	0.000	0.002
*Bacteroides coprosuis*	−1.68	0.000	0.002
*Phocaeicola coprophilus*	−1.87	0.000	0.008
Candidatus Arthromitus sp. SFB rat Yit	0.84	0.000	0.039
*Prevotella jejuni*	−1.31	0.000	0.039
*Prevotella intermedia*	−1.35	0.000	0.047
*Prevotella nigrescens*	−1.38	0.000	0.047
*Propionibacterium australiense*	0.74	0.001	0.078
*Prevotella fusca*	−1.28	0.001	0.078
*Prevotella oris*	−1.31	0.001	0.078
*Streptococcus sobrinus*	−1.39	0.001	0.078
*Paraprevotella xylaniphila*	−1.98	0.001	0.078
Prevotella sp. WR041	−1.31	0.001	0.078
*Rhodopseudomonas palustris*	0.65	0.001	0.078
*Paludibacterium* sp. B53371	0.75	0.001	0.078
*Nitrogeniibacter mangrovi*	0.76	0.001	0.078
*Prevotella enoeca*	−1.19	0.002	0.094
*Parabacteroides distasonis*	−1.49	0.002	0.094

Differentially abundant taxa between RA patients who achieved minimum clinically important improvement (MCII+) and who did not (MCII-) during the first six weeks of DMARD treatment. Linear contrasts have been constructed and tested for significance using voom: limma approach combined with GMPR normalisation. Adjustment for multiple testing was done using Benjamini–Hochberg false discovery rate within each taxonomic level. Reported are findings with FDR <10% in at least one contrast. No significant results were obtained for comparisons between MCII+ and MCII- at baseline.

*Prevotella* are commonly associated with RA pathogenesis. In humans, these commensal microbes colonise the skin, vagina, oral cavity, respiratory tract and intestine, and have important roles in metabolism and health [29]. Here we add further evidence in support of gut *Prevotella* spp. involvement in RA prognosis, by contrasting MCII+ and MCII-. After six weeks of DMARD treatment, 18 microbes significantly differed in patients’ gut microbiome showing clinical response to therapy, of those 13 decreased including seven *Prevotella* spp. (see Table 4). Furthermore, 15 *Prevotella* spp. were identified to decline within long-term DMARD-treated NOAR participants, when compared with early-onset DMARD-naive patients. In total, 15 different *Prevotella* spp. were negatively associated with clinical improvement to therapy, including *P. denalis, P. denticola, P. histicola, P. intermedia, P. melaninogenica, P. nigrescens, P. oris* and *P. ruminicola.* These species have been linked to respiratory disease, multiple sclerosis, brain abscess, blood and cardiac infection, musculoskeletal infection, and most commonly periodontal disease [29]. Previously, we reported *Prevotella* spp. play a role in RA development, and microbial abundance increased in those with the genotype predisposing risk for RA even in the absence of disease [5]. Interestingly, we did not detect significant associations between *P. copri*, DMARD treatment and patient clinical response. It is well established that *P. copri* is involved in early RA, with increased abundance thought to stimulate expression of anti-citrullinated proteins and serum antibodies [6, 8]. Genetic factors predispose to RA, with additive influence of acquired environmental risk [30]. Host genetic factors mediate the microbiome and are thought to lead to a pro-inflammatory, hyper-active immune type [5, 30]. The presence of anti-CCP antibodies at baseline may be considered to represent the shared epitope risk, in line with our previous study demonstrating increased *Prevotella spp.* in those positive for the shared epitope [5]. Regression models for stool including anti-CCP antibody status as covariate showed no influence on alpha-diversity in our sample. We identified seven *Prevotella spp.* to be significantly elevated in those having anti-CCP antibodies compared with those without, and several other species including *P. copri* showed a similar trend (Supplementary Table 12, available at *Rheumatology* online) [31].

Of metagenomics studies investigating RA and the microbiome; two reported taxa differences between participants who respond well to DMARD therapies compared with those who do not [12, 13]. In line with our findings, no differential abundance in *P. copri* was reported by either group. Whilst a common theme across RA literature, we speculate that abundance of *P. copri* is more prominent when comparing healthy and disease participants, which may explain the lack of association in this study. *P. copri* comprises four genetically distinct clades with genetic and functional differences that are influenced by diet and lifestyle [32–34]. For example, in plant-rich ‘non-westernised’ diets, *P. copri* can catabolise plant carbohydrates and fibres; alternatively, in higher protein diets *P. copri* is known to break down branch chain amino acids (BCAAs) from meats [29, 32, 34]. Interestingly, in a recent study gut isolates of *P. copri* strains were shown to differ in RA participants, with more severe arthritis induced in mice from RA patient strains than from healthy controls [34]. These findings warrant further investigation and may extend more broadly to *Prevotella spp.* Of note, periodontal disease appears to commonly be associated with the *Prevotella* spp. we identified (e.g. *P. denalis, P. denticola, P. histicola, P. intermedia, P. melaninogenica, P. nigrescens, P. oris, P. ruminicola*) with poor treatment outcome [29].

Periodontal disease is more frequent in RA [35]. Despite environmental niches and segregation, it is common for microbes to translocate from the oral cavity to the gut. Common examples in RA are *Porphyromonas gingivalis* and *P. copri*; however, *Streptoccocus* spp. are also associated with oral to gut translocation [36]. In the oral cavity, streptococci are known to form early-plaque biofilms that can support microbes associated with gingival disease, such as *P. gingivalis* [37]. Intriguingly, *P. gingivalis* was identified in higher abundance in baseline samples when compared with long-term DMARD participants in NOAR. Furthermore *Streptococcus* spp. significantly decreased in the gut of early RA patients having MCII+ after 12 weeks of DMARD treatment, in this study.

Our findings support the hypothesis of DMARD restoration of a eubiotic gut microbiome when patient and treatment align [38, 39]. We had anticipated finding baseline microbiome samples predictive of response to treatment. While baseline differential abundance analysis did not discriminate responders (MCII+) from non-responders (MCII-), longitudinal analysis showed changing microbiota and positive response to DMARDs. At 6 weeks there was a reduction of multiple species of *Prevotella* in responsive participants. At 12 weeks, multiple species of Streptococci were reduced in responders. Methotrexate was associated with beta-diversity difference following six weeks of treatment, even when adjusting for disease activity and other confounders (age, sex, BMI, smoking status, ethnicity, hospital centre, diet and other DMARDs).

Predictive tools for RA treatment outcome utilising oral and gut microbiome are advancing [12–14]. In a small Hispanic cohort (*n* = 26) a model using DMARD-naive gut metagenomes from patients receiving monotherapy methotrexate was able to predict non-responsiveness [12]. Gupta and colleagues [13] retrospectively used gut metagenomes from patients (*n* = 32) taking DMARDs to predict MCII response with 90% accuracy using a neural network. Similarly, Zhang and colleagues [14] accurately predicted DAS28-ESR response in using long-term DMARD-treated patients and controls, with dental metagenome samples. Our models used gut and oral metagenomes to predict DMARD-naive MCII response. Although our models were not as strong as those cited above, we were able to better identify MCII+ as highlighted by our sensitivity scores (Supplementary Tables 13–15, available at *Rheumatology* online). Northern Europeans accounted for >60% of our sample and patients often received combination therapy to treat RA. The real strength of our work lies in our validation using two external data cohorts, a novel and key contrast to similar studies that may have overfit models with small sample sizes and a lack of external validation. Another strength is that the population cohort of DMARD-naive participants permits the exploration of the influence of therapy.

The range of DAS28 values at baseline was large, although the lower quantile cut-off at baseline was 3.6, therefore comprising those with clinically meaningful disease activity. When low DAS scores were removed from the analysis, the negative linear relationship with alpha diversity was maintained. This association decreased with received treatment; for example, DAS28 negatively associated with alpha-diversity at baseline, although an improvement in DAS28 is seen in patients at six (48.2%) and 12 weeks (50%) (Table 1) following DMARD treatment. Interestingly, when broken down the tender joint score was the principal DAS28 measure that negatively associated with alpha-diversity.

Long-term DMARD comparison came from NOAR: study participants registered to assist arthritis research. Disease activity was from medical records and was participant generated, not clinician scored, so we used available data to calculate DAS28-CRP. Differential abundance analysis in NOAR using the criteria cut-point of DAS28-CRP <2.6 showed one taxa altered, *Actinomyces sp. Oral taxon 414*, between high- *vs* low disease activity (Supplementary Table 11, available at *Rheumatology* online). Furthermore, the predictive model used in the drug-naive cohort resulted in similar AUC for gut microbiota at 0.65 AUC (Supplementary Table 13, available at *Rheumatology* online).

We acknowledge that there are limitations to this study. Changes in saliva metagenomes were sparse, with smoking status and the predictive model with metacyc pathways being the only clear signals achieved. Similar predictive results were seen in Zhang and colleagues when examining saliva; however, dental microbiota were able to predict response [14]. The longitudinal data collection was for 12 weeks post recruitment, though longer follow-up of 6–12 months would have been preferable.

A strength of our work is the ethnic mix recruited to the study reflecting an inner-city UK sample: we found beta-diversity to be reduced in northern Europeans compared with south Asians at baseline, likely driven by the small sub-group numbers, but there was no impact of ethnicity on predictive models. Interestingly, *Prevotella* spp. are known to dominate the gut microbiome in ‘non-westernised’ populations [29]. The interplay between ethnicity, the microbiome and RA warrants investigation and may give further insight into patient-specific treatment.

In summary, we identified a partial restoration of the microbiome to a more eubiotic state in RA patients at 6 weeks and 12 weeks DMARD treatment in participants that responded well to DMARD therapy. This was further supported by long-term (>1 year) treated DMARD RA participants with similar community shifts. Finally, microbiomes provide a promising diagnostic tool for guiding therapeutic decisions in future.

## Supplementary Material

keae045_Supplementary_Data

## Data Availability

The data underlying this article are available in Sequence Read Archive (SRA) at https://dataview.ncbi.nlm.nih.gov/object/PRJNA957107?reviewer=e4pj6rso2m8osj2c6hilgqn9ei, and can be accessed with accession number PRJNA957107.
