# Supplementary material for: Treatment response in rheumatoid arthritis is predicted by the microbiome: a large observational study in UK DMARD-naive patients
Source: Rheumatology (Oxford). 2024 Jan 30;63(12):3486–95. doi: 10.1093/rheumatology/keae045 (PMC11637416; doi:10.1093/rheumatology/keae045)
Supplement: keae045_Supplementary_Data [file keae045_supplementary_data.docx]

Table of Contents:

[**Supplementary Methods:** 3](#_Toc145760579)

[**Clinical Assessments and Demographics:** 3](#_Toc145760580)

[**i) Study numbers** 3](#_Toc145760581)

[**ii) Medical Data Form** 3](#_Toc145760582)

[**iii) Microbiome Study General Questionnaire (MSGQ) and Food Frequency Questionnaire (FFQ)** 3](#_Toc145760583)

[**vi) Clinical Microbiome Health Questionnaire (CMHQ)** 3](#_Toc145760584)

[**Sample collection and shotgun metagenomic processing:** 3](#_Toc145760585)

[**i) Stool and saliva collection** 3](#_Toc145760586)

[**ii) DNA extraction: Stool and saliva samples** 3](#_Toc145760587)

[**iii) Shotgun metagenomics: DNA sequencing from stool and saliva** 4](#_Toc145760588)

[**iv) Differential abundance analysis** 4](#_Toc145760589)

[**References:** 5](#_Toc145760590)

[**Supplementary results:** 6](#_Toc145760591)

**List of Tables:**

[**Supplementary Table 1:** Clinical and demographic characteristics of Norfolk Arthritis Register (NOAR) RA participants 6](#_Toc153274754)

[**Supplementary Table 2:** Disease activity and microbiome alpha-diversity in saliva 7](#_Toc153274755)

[**Supplementary Table 3:** The contribution of RA patient characteristics to saliva microbiome variance 8](#_Toc153274756)

[**Supplementary Table 4:** Differential abundance analysis of the gut microbiota between baseline and follow-up 9](#_Toc153274757)

[**Supplementary Table 5**: Differential abundance analysis of the saliva microbiota between baseline and follow-up 10](#_Toc153274758)

[**Supplementary Table 6:** Differentially abundant taxa in saliva microbiota between RA patients who achieved minimum clinically important improvement (MCII+) at baseline and six weeks treatment 23](#_Toc153274759)

[**Supplementary Table 7:** Differentially abundant taxa in saliva microbiota between RA patients who did not achieve minimum clinically important improvement (MCII-) at baseline and 12 weeks treatment 29](#_Toc153274760)

[**Supplementary Table 8:** Differentially abundant stool microbiota between RA patients who achieved minimum clinically important improvement (MCII+) between baseline and 12 weeks treatment 31](#_Toc153274761)

[**Supplementary Table 9:** Differentially abundant taxa in stool microbiota between short-term DMARD-treated RA patients (IMRABIOME) at baseline and long-term DMARD-treated RA (NOAR) 36](#_Toc153274762)

[**Supplementary Table 10:** Differentially abundant taxa in stool microbiota between short-term DMARD-treated RA patients (IMRABIOME) at follow-up and long-term DMARD-treated RA (NOAR). 38](#_Toc153274763)

[**Supplementary Table 11:** Differential abundance of responders vs non-responders in the NOAR cohort 39](#_Toc153274764)

[**Supplementary Table 12:** Increased abundance of Prevotella species in anti-CCP positive patients at baseline compared to anti-CCP negative 40](#_Toc153274765)

[**Supplementary Table 13:** Confusion matrices of stool microbiota, metacyc pathways and KO genes in baseline DMARD-naive patients 41](#_Toc153274766)

[**Supplementary Table 14:** Confusion matrices of saliva microbiota, metacyc pathways and KO genes in baseline DMARD-naive patients 42](#_Toc153274767)

[**Supplementary Table 15:** Confusion matrices of NOAR stool and saliva microbiota, metacyc pathways and KO genes in long-term DMARD-treated patients 43](#_Toc153274768)

# **Supplementary Methods:**

## **Clinical Assessments and Demographics:**

### **i) Study numbers**

144 participants were recruited to this study at baseline sample collection. In total, 23 participants withdrew from the study and a further 20 did not participate in follow-up sample collections – between baseline and six weeks 18 participants withdrew and 11 were lost to follow-up, and between six and 12 weeks five withdrew and nine were lost to follow-up.

### **ii) Medical Data Form** (Baseline, six and twelve weeks follow-up)

We utilised disease activity evaluations which are administered as part of standard clinical practice including the Disease Activity Score for 28 Joints (DAS28), Simplified Disease Activity Index (SDAI) and Clinical Disease Activity Index (CDAI) which all measure swelling and tenderness in joints and global well-being from the point of view of patient (DAS28), clinician (CDAI) or both (SDAI).

Standard clinical care records were accessed to ascertain measures of rheumatoid factor (RF), ACPA, erythrocyte sedimentation rate (ESR) and C-reactive protein (CRP). Full blood count (FBC), liver function tests (LFTs) and X-ray reports of chest, hands and feet were searched and recorded at baseline and both follow-up time points. Current and planned medications and doses were noted including the prescription of DMARDs (methotrexate, hydroxychloroquine and sulfasalazine).

### **iii) Microbiome Study General Questionnaire (MSGQ) and Food Frequency Questionnaire (FFQ)** (Baseline, six and twelve weeks follow-up)

The MSGQ identifies factors likely to influence the microbiome. The histories of antimicrobial medication use such as antibiotics, gastrointestinal diseases and supplement consumption, current diet and food allergies were recorded. The dietary information was collected following the Food Frequency Questionnaire commonly used and adapted at Twins UK [1]. The Bristol Stool Chart was provided for participants to self-identify the type of sample they provided.

### **vi) Clinical Microbiome Health Questionnaire (CMHQ)** (Baseline)

The CMHQ includes questions about general participant demographics such as gender, age, ethnicity, education, height and weight. From the latter two measures BMI was calculated (kg/m2). Lifestyle factors such as diet, physical activity, alcohol, and smoking consumption were recorded. A detailed medical history was taken including frequency and severity of illnesses, fractures, urology, periodontal disease and dentistry. Recent access of medical care was recorded and independent living assessed along with questions about current and recent experiences and locations of joint and muscular pain (e.g., was pain experienced ‘in the neck and back’ or ‘the arms and hands’ or ‘the legs and feet’… ‘during the last three months.’).

# **Sample collection and shotgun metagenomic processing:**

### **i) Stool and saliva collection**

At baseline participants were asked to provide stool and saliva samples prior to commencing their DMARD medication. Saliva samples were collected in 5mL sterile pots, on the day of the baseline clinical visit Participants were provided with a stool sample kit that fully detailed the instructions on preparing and collecting the sample and were stored with a purpose designed icepack, until specialised postage and delivery the following day. Samples were either mailed from the participant to the lab in special mailing boxes or were collected by research nurses/assistants at follow up appointments and couriered to the laboratory. Samples were received and stored in -80°C within 24 hours of being collected and remained in -80° C storage until processing. Sample collection date, arrival date at the laboratory and stool weighed were recorded prior to processing and storage.

### **ii) DNA extraction: Stool and saliva samples**

**Stool:**

To isolate genomic DNA (gDNA) from faecal material stool was processed prior to storage in 2 x 25mL Bijou tubes. Prior to gDNA extraction, samples were thawed on ice for an hour or in the fridge at 4° C. gDNA was washed in a two-step process and eluted in MagMax Core Elution Buffer. This method is a modification of the manufacturers protocol, optimised for 1g starting weight of faeces, using the Magmax CORE mechanical Lysis module (Kit catalogue no. A37487).

In short, stool material is mechanically lysed with 2 x 5mm core glass beads for 10 seconds in a Spex Frinder at a setting rate of 800, and material is left to settle. 1mL of supernatant was transferred to a 2mL Core beat tubes and centrifuged for 10 minutes at 15,000g. Supernatant was removed and 400µL Core Clarifying solution added to the tubes. The Spex Grinder was then used to bead beat the samples for 5 minutes at a rate of 1000, following this they were centrifuged for 3 minutes. 10µL PK solution was added to 300µL of lysate for each sample and rested before 720µL of Lysis/Bind Master Mix was added. The KingFisher automated processing was conducted following the SOP-King Fisher Flex. gDNA extracted from samples were then stored at -80° C until shotgun metagenomic sequencing was undertaken by Clinical Microbiomics.

**Saliva:**

A 500µL aliquot of saliva was sent to Clinical Microbiomics for DNA. gDNA was extracted from the samples using NucleoSpin® 96 Soil (Macherey-Nagel). Bead beating was done on a Vortex-Genie 2 horizontally at 2700rpm for 5 minutes. A minimum of one positive control (ZymoBIOMICS™ Microbial Community Standard, Zymo Research) and one negative control was included with each batch of samples. The following sample input weight/volume was used: 250µL of saliva.

### **iii) Shotgun metagenomics: DNA sequencing from stool and saliva**

**Clinical Microbiomics methodology:**

Prior to sequencing, gDNA was quality and quantity assessed by agarose gel electrophoresis and Qubit 2.0 fluorometer for each sample. gDNA was randomly sheared into fragments of around 350 base pairs in size. The fragmented gDNA was used for library construction using NEBNext Ultra Library Prep Kit for Illumina (New England Biolabs). The prepared gDNA libraries were evaluated using Qubit 2.0 fluorometer quantitation and Agilent 2100 Bioanalyzer for the fragment size distribution. Quantitative real-time PCR (qPCR) was used to determine the concentration of the final library before sequencing. The library was sequenced using 2 × 150 bp paired-end sequencing on an Illumina NovaSeq 6000 platform.

Raw sequencing data was returned from Clinical Microbiomics and was pre-processed using the default parameters of YAMP, a containerised metagenomic workflow. [2] The pipeline removes sequencing adapter, primer, duplicates and low-quality sequences from raw sequencing data. Trimmed reads shorter than 100 bases were discarded as low-quality reads. Sequence reads that map to the human genome (GRCh38) were discarded using default features of Bowtie2 (v0.2.3.2), [3] and read pairs in which both reads passed filtering were retained. Taxonomic profiling was completed using kracken2/bracken, [4] while HUMAnN3 [5] was used for function profiling with UniRef90 reference database within the YAMP framework. Prior to microbiome analyses, viral and eukaryotic data were removed, leaving archaea and bacteria for subsequent analysis.

### **iv) Differential abundance analysis**

Comparison of different taxonomic abundances between time points and clinical data were normalised using GMPR (v0.1.3) [7] and carried out using the limma:voom pipeline (v3.52.4) [8, 9]. Statistical significance threshold was set at 10% false discovery rate (FDR). To determine if baseline microbiome data can predict DMARD treatment response, Clinical Disease Activity Index (CDAI) score was used to classify patients into two groups according to minimal clinically important improvement (MCII), as defined in Gupta and colleagues [5]. This allowed the comparison of those with a meaningful clinical response to DMARD therapy with participants that did not.

Differential abundance analysis was carried out:

- at baseline, between MCII+/MCII- groups as established at follow-up,
- at follow-up, between MCII+/MCII- groups,
- between MCII+ at baseline and follow-up, and
- between MCII- at baseline and follow-up.

This design tested the association between the microbiome and MCII as measured by changes in CDAI score; to test if the microbiome at baseline was predictive of MCII at follow-up; and to assess microbiome dynamics among those who achieved MCII and those who did not.

# **References:**

1. Bingham S. Validation of dietary assessment methods in the UK arm of EPIC using weighed records, and 24-hour urinary nitrogen and potassium and serum vitamin C and carotenoids as biomarkers. International Journal of Epidemiology. 1997 Feb 1;26(90001):137S151.
2. Visconti A, Martin TC, Falchi M. YAMP: a containerized workflow enabling reproducibility in metagenomics research. GigaScience 2018;7(7).
3. Langmead B, Salzberg SL. Fast gapped-read alignment with Bowtie 2. Nature Methods 2012;9(4):357-9.
4. Wood DE, Lu J, Langmead B. Improved metagenomic analysis with Kraken 2. Genome Biology 2019;20(1):257.
5. Beghini F, McIver LJ, Blanco-Míguez A, et al. Integrating taxonomic, functional, and strain-level profiling of diverse microbial communities with bioBakery 3. eLife 2021;10:e65088.
6. Gupta VK, Cunningham KY, Hur B, et al. Gut microbial determinants of clinically important improvement in patients with rheumatoid arthritis. Genome Med 2021;13(1):149.
7. Chen L, Reeve J, Zhang L, Huang S, Wang X, Chen J. GMPR: A robust normalization method for zero-inflated count data with application to microbiome sequencing data. PeerJ 2018;6:e4600.
8. Law CW, Chen Y, Shi W, Smyth GK. voom: precision weights unlock linear model analysis tools for RNA-seq read counts. Genome Biology 2014;15(2):R29.
9. Ritchie ME, Phipson B, Wu D, et al. limma powers differential expression analyses for RNA-sequencing and microarray studies. Nucleic Acids Research 2015;43(7):e47-e.

# **Supplementary results:**

**Supplementary Table 1:** Clinical and demographic characteristics of Norfolk Arthritis Register (NOAR) RA participants

| **Trait** | | **NOAR , n = 165** |
| --- | --- | --- |
| **Sex** | Female, n (%) | 126 (76·4) |
|  | Male, n (%) | 39 (23·6) |
|  |  |  |
| **Age, mean±SD** |  | 53·4 ± 11.78 |
|  |  |  |
| **Smoking status** | Never, n (%) | 71 (43·2) |
|  | Ever, n (%) | 84 (50·1) |
|  | Current, n (%) | 10 (6·7) |
|  |  |  |
| **Body mass index, mean±SD** |  | 27.06 ± 5.5 |
|  |  |  |
| **Ethnicity** | Northern European, n (%) | 163 (98.7) |
|  | Other, n (%) | 2 (1.3) |
|  |  |  |
| **DAS28-CRP score** | Median (min,max) | 3.44 (1.2;7.2) |
|  | High, n (%) | 22 (13.3) |
|  | Moderate, n (%) | 72 (43.6) |
|  | Low, n (%) | 71 (43.0) |
|  |  |  |
| **DMARD Treatment** |  |  |
| **Total use (any)** | Methotrexate, n (%) | 123 / 165 (74.5)  ) |
|  | Sulfasalazine, n (%) | 15 / 165 (9.0) |
|  | Hydroxychloroquine, n (%) | 50 / 165 (30.3) |

Legend to Supplementary Table 1: DAS28-CRP was a patient evaluated score, it was not scored by a clinical, RA = rheumatoid arthritis, BMI = body mass index, DAS = Disease Activity Score, SD = standard deviation

**Alpha-diversity:**

**Supplementary Table 2:** Disease activity and microbiome alpha-diversity in saliva

| **Trait** | **Model** | **Baseline** | **Six weeks** | **Twelve weeks** |
| --- | --- | --- | --- | --- |
| **Chao1** | Smoking | β = 22·6 ± 77·9; p = 0·77 | β = -21·2 ± 78·3; p = 0·78 | β = -3·45 ± 100; p = 0·97 |
|  | DAS28 | β = -38·2 ± 75·0; p = 0·61 | β = 71·6 ± 86·4; p = 0·40 | β = -138·1 ± 110; p = 0·21 |
|  | CDAI | β = 3·41 ± 7·31; p = 0·64 | β = -3·87 ± 9·91; p = 0·69 | β = 19·8 ± 12·5; p = 0·11 |
| **Shannon** | Smoking | β = -0·09 ± 0·04; p = 0·04 | β = -0·10 ± 0·045; p = 0·02 | β = -1·58 ± 0·061; p = 0·01 |
|  | DAS28 | β = -0·03 ± 0·04; p = 0·41 | β = 0·02± 0·05; p = 0·67 | β = 1·28 ± 0·067; p = 0·85 |
|  | CDAI | β = 0·002 ± 0·004; p = 0·56 | β = -0·003± 0·005; p = 0·49 | β = 5·02 ± 0·007; p = 0·94 |
| **Simpson** | Smoking | β = -1·25 ± 0·004; p = 0·01 | β = -0·008 ± 0·004;p = 0·06 | β = -1·34 ± 0·006; p = 0·04 |
|  | DAS28 | β = -1·18 ± 0·004; p = 0·80 | β = 0·0004 ± 0·004;p = 0·38 | β = 8·54 ± 0·007; p = 0·90 |
|  | CDAI | β = 1·08 ± 0·0004; p = 0·98 | β = -0·0004 ± 0·0005;p = 0·42 | β = 1·94± 0·0008; p = 0·81 |

Legend to Table 2: Estimates ± standard error and p-values for multiple regression of RA activity scores on Chao1, Shannon and Simpson indices; adjusted for age, sex, BMI, and smoking status. RA = rheumatoid arthritis, BMI = body mass index, DAS = Disease Activity Score, CDAI = Clinical Disease Activity Index.

**Beta-diversity:**

**Supplementary Table 3:** The contribution of RA patient characteristics to saliva microbiome variance

| **Trait** | **Model** | **Baseline** | | **Follow up 1** | | **Follow up 2** | |
| --- | --- | --- | --- | --- | --- | --- | --- |
|  |  | R^2^ | p-value | R^2^ | p-value | R^2^ | p-value |
| **Age** | Univariate | 1·8 | 0·0076 | 1·7 | 0·0590 | 2·0 | 0·0470 |
|  | Adjusted | 1·6 | 0·0168 | 1·7 | 0·0745 | 2·0 | 0·0784 |
| **Sex** | Univariate | 0·7 | 0·3210 | 1·2 | 0·1984 | 1·3 | 0·2512 |
|  | Adjusted | 0·6 | 0·5443 | 1·3 | 0·1572 | 1·5 | 0·2042 |
| **BMI** | Univariate | 0·9 | 0·1451 | 1·4 | 0·1249 | 2·6 | 0·0187 |
|  | Adjusted | 0·7 | 0·3643 | 1·2 | 0·2158 | 1·7 | 0·1340 |
| **Smoking** | Univariate | 3·7 | 0·0007 | 4·7 | 0·0023 | 4·7 | 0·0051 |
|  | Adjusted | 3·5 | 0·0023 | 4·5 | 0·0053 | 4·7 | 0·0114 |
| **Site (hospital)** | Univariate | 2·3 | 0·1968 | 4·0 | 0·0717 | 5·7 | 0·0156 |
|  | Adjusted | 2·2 | 0·3634 | 3·9 | 0·1106 | 5·3 | 0·0534 |
| **Ethnicity** | Univariate | 4·6 | 0·0908 | 5·9 | 0·1828 | 4·6 | 0·8606 |
|  | Adjusted | 3·2 | 0·6298 | 5·0 | 0·3709 | 5·0 | 0·7146 |
| **DAS28 score** | Univariate | 1·0 | 0·0934 | 1·0 | 0·2954 | 1·1 | 0·4567 |
|  | Adjusted | 0·6 | 0·5250 | 0·7 | 0·6250 | 1·4 | 0·2645 |
| **Methotrexate – mono** | Univariate | - | - | 2·2 | 0·2143 | 2·6 | 0·2661 |
|  | Adjusted | - | - | 2·0 | 0·3006 | 3·1 | 0·3276 |
| **Sulfasalazine – mono** | Univariate | - | - | 1·5 | 0·1804 | 1·2 | 0·4439 |
|  | Adjusted | - | - | - | - | - | - |
| **Hydroxychloroquine– mono** | Univariate | - | - | 3·0 | 0·0758 | 3·1 | 0·1476 |
|  | Adjusted | - | - | - | - | - | - |
| **Use of methotrexate** | Univariate | - | - | 1·0 | 0·2753 | 1·4 | 0·1973 |
|  | Adjusted | - | - | 0·7 | 0·5617 | 1·8 | 0·1155 |
| **Use of sulfasalazine** | Univariate | - | - | 1·2 | 0·1671 | 1·8 | 0·0840 |
|  | Adjusted | - | - | 0·9 | 0·4405 | 1·0 | 0·4906 |
| **Use of hydroxychloroquine** | Univariate | - | - | 1·7 | 0·0581 | 1·2 | 0·3364 |
|  | Adjusted | - | - | 1·4 | 0·1153 | 1·4 | 0·2625 |

Legend to table 3: Bray-Curtis dissimilarity was calculated using saliva microbiome at species level; because of high correlation between DAS28 and CDAI scores, we chose to analyse DAS28 score only. BMI = body mass index, DAS28 = Disease Activity Score 28, CDAI = Clinical Disease Activity Index, mono = monotherapy.

**Differential abundance:**

**Supplementary Table 4:** Differential abundance analysis of the gut microbiota between baseline and follow-up

| **Taxonomic level** | **Taxa** | **Six weeks vs Baseline** | | | **12 weeks vs Baseline** | | |
| --- | --- | --- | --- | --- | --- | --- | --- |
|  |  | **logFC** | **p-value** | **adj. p-value** | **logFC** | **p-value** | **adj. p-value** |
| **Family** | Blattabacteriaceae | -0.30 | 0.001 | 0.142 | -0.43 | 1.9E-05 | 0.002 |
|  | Oscillospiraceae | 0.22 | 0.035 | 0.799 | 0.36 | 0.002 | 0.083 |
|  | Fusobacteriaceae | -0.07 | 0.475 | 0.819 | -0.33 | 0.002 | 0.083 |
| **Genus** | Blattabacterium | -0.31 | 8.1E-04 | 0.135 | -0.42 | 1.9E-05 | 0.006 |
|  | Pradoshia | -0.22 | 0.037 | 0.846 | -0.43 | 1.6E-04 | 0.027 |
|  | Paraclostridium | -0.39 | 0.005 | 0.362 | -0.53 | 4.6E-04 | 0.041 |
|  | Crassaminicella | -0.21 | 0.008 | 0.454 | -0.30 | 4.9E-04 | 0.041 |
|  | Faecalibacterium | 0.34 | 0.013 | 0.556 | 0.48 | 0.001 | 0.070 |
|  | Paeniclostridium | -0.27 | 0.038 | 0.846 | -0.46 | 0.001 | 0.070 |
|  | Fusobacterium | -0.08 | 0.425 | 0.898 | -0.34 | 0.002 | 0.083 |
| **Species** | *Streptococcus mutans* | -1.07 | 1.9E-06 | 0.001 | -0.95 | 7.5E-05 | 0.024 |
|  | *Blattabacterium cuenoti* | -0.30 | 9.3E-04 | 0.144 | -0.42 | 2.1E-05 | 0.016 |
|  | *Thermoanaerobacterium sp. RBIITD* | -0.26 | 0.003 | 0.280 | -0.36 | 1.2E-04 | 0.024 |
|  | *Crassaminicella thermophila* | -0.24 | 0.005 | 0.296 | -0.35 | 1.3E-04 | 0.024 |
|  | *Pradoshia sp. D12* | -0.23 | 0.028 | 0.573 | -0.43 | 1.6E-04 | 0.024 |
|  | *Paraclostridium bifermentans* | -0.36 | 0.011 | 0.360 | -0.51 | 8.5E-04 | 0.093 |
|  | *Bacillus mycoides* | -0.17 | 0.073 | 0.783 | -0.35 | 7.3E-04 | 0.093 |

**Legend to Table 4:** Linear contrasts have been constructed and tested for significance using voom:limma approach combined with GMPR normalization. Adjustment for multiple testing was done using Benjamini-Hochberg false discovery rate withing each taxonomic level. Reported are findings with FDR <10% in at least one contrasts. No significant results were obtained for comparisons between follow-up time points.

**Supplementary Table 5**: Differential abundance analysis of the saliva microbiota between baseline and follow-up

| **Taxonomic level** | **Taxa** | **12 weeks vs Baseline** | | |
| --- | --- | --- | --- | --- |
|  |  | **logFC** | **p-value** | **adj. p-value** |
| Phylum | Fusobacteria | 0.4800 | 0.0108 | 0.0455 |
|  | Bacteroidetes | 0.3900 | 0.0207 | 0.0455 |
|  | Firmicutes | 0.4700 | 0.0204 | 0.0455 |
|  | Verrucomicrobia | 0.4800 | 0.0175 | 0.0455 |
|  | Actinobacteria | 0.4600 | 0.0195 | 0.0455 |
|  | Spirochaetes | 0.6500 | 0.0280 | 0.0510 |
|  | Candidatus.Saccharibacteria | 0.6000 | 0.0324 | 0.0510 |
|  | Proteobacteria | 0.4100 | 0.0379 | 0.0521 |
|  | Synergistetes | 0.6000 | 0.0789 | 0.0965 |
| Family | Sanguibacteraceae | 0.7700 | 0.0000 | 0.0022 |
|  | Bogoriellaceae | 0.6600 | 0.0005 | 0.0305 |
|  | Beutenbergiaceae | 0.6700 | 0.0008 | 0.0305 |
|  | Xanthomonadaceae | 0.7100 | 0.0006 | 0.0305 |
|  | NA.9 | 0.6100 | 0.0019 | 0.0360 |
|  | Polyangiaceae | 0.5500 | 0.0019 | 0.0360 |
|  | Cellulomonadaceae | 0.6300 | 0.0012 | 0.0360 |
|  | Streptomycetaceae | 0.6200 | 0.0017 | 0.0360 |
|  | Rhizobiaceae | 0.6100 | 0.0021 | 0.0365 |
|  | Streptosporangiaceae | 0.5500 | 0.0026 | 0.0408 |
|  | Vallitaleaceae | 0.5800 | 0.0036 | 0.0503 |
|  | Nocardiaceae | 0.6100 | 0.0045 | 0.0507 |
|  | Micromonosporaceae | 0.5400 | 0.0045 | 0.0507 |
|  | Dermabacteraceae | 0.5600 | 0.0046 | 0.0507 |
|  | Acidaminococcaceae | 0.6000 | 0.0057 | 0.0553 |
|  | Oscillospiraceae | 0.4600 | 0.0055 | 0.0553 |
|  | Eubacteriales Family XIII Incertae Sedis | 0.5500 | 0.0065 | 0.0585 |
|  | Eggerthellaceae | 0.7000 | 0.0071 | 0.0610 |
|  | Clostridiaceae | 0.4800 | 0.0089 | 0.0639 |
|  | Nocardioidaceae | 0.5700 | 0.0082 | 0.0639 |
|  | Akkermansiaceae | 0.4700 | 0.0090 | 0.0639 |
|  | Veillonellaceae | 0.5600 | 0.0091 | 0.0639 |
|  | NA.35 | 0.8900 | 0.0101 | 0.0650 |
|  | Paenibacillaceae | 0.4300 | 0.0101 | 0.0650 |
|  | Alcaligenaceae | 0.5100 | 0.0120 | 0.0691 |
|  | Eubacteriaceae | 0.5700 | 0.0130 | 0.0691 |
|  | Promicromonosporaceae | 0.4800 | 0.0128 | 0.0691 |
|  | Coriobacteriaceae | 0.5100 | 0.0124 | 0.0691 |
|  | Geodermatophilaceae | 0.5500 | 0.0121 | 0.0691 |
|  | NA.11 | 0.5000 | 0.0141 | 0.0726 |
|  | Thermomonosporaceae | 0.4600 | 0.0153 | 0.0734 |
|  | NA.34 | 0.8600 | 0.0152 | 0.0734 |
|  | Erysipelotrichaceae | 0.4200 | 0.0163 | 0.0734 |
|  | Intrasporangiaceae | 0.4900 | 0.0167 | 0.0734 |
|  | Nocardiopsaceae | 0.5100 | 0.0164 | 0.0734 |
|  | Campylobacteraceae | 0.5200 | 0.0178 | 0.0763 |
|  | Ruaniaceae | 0.4800 | 0.0185 | 0.0769 |
|  | Brevibacteriaceae | 0.4600 | 0.0190 | 0.0769 |
|  | Ornithinimicrobiaceae | 0.5000 | 0.0204 | 0.0805 |
|  | Kytococcaceae | 0.5300 | 0.0209 | 0.0805 |
|  | Actinomycetaceae | 0.4600 | 0.0221 | 0.0810 |
|  | Bacillaceae | 0.3600 | 0.0217 | 0.0810 |
|  | Lachnospiraceae | 0.4100 | 0.0238 | 0.0851 |
|  | Pectobacteriaceae | 0.4300 | 0.0244 | 0.0854 |
|  | Dietziaceae | 0.4800 | 0.0279 | 0.0923 |
|  | NA.36 | 0.7600 | 0.0297 | 0.0923 |
|  | Prevotellaceae | 0.4200 | 0.0291 | 0.0923 |
|  | Dermacoccaceae | 0.4600 | 0.0300 | 0.0923 |
|  | Atopobiaceae | 0.5000 | 0.0297 | 0.0923 |
|  | NA.20 | 0.7100 | 0.0283 | 0.0923 |
|  | Propionibacteriaceae | 0.5300 | 0.0320 | 0.0967 |
|  | Dermatophilaceae | 0.4100 | 0.0328 | 0.0971 |
| Genus | Sanguibacter | 0.8000 | 0.0000 | 0.0028 |
|  | Anaerocolumna | 0.9000 | 0.0000 | 0.0028 |
|  | Isoptericola | 0.8600 | 0.0001 | 0.0072 |
|  | Nonomuraea | 0.7200 | 0.0002 | 0.0113 |
|  | Erysipelatoclostridium | 0.7600 | 0.0002 | 0.0113 |
|  | Anaerotignum | 0.7800 | 0.0002 | 0.0113 |
|  | Micropruina | 0.8300 | 0.0002 | 0.0113 |
|  | Oerskovia | 0.7800 | 0.0004 | 0.0151 |
|  | Lachnoclostridium | 0.7000 | 0.0004 | 0.0151 |
|  | Acidipropionibacterium | 0.6900 | 0.0004 | 0.0152 |
|  | Nocardia | 0.7500 | 0.0006 | 0.0162 |
|  | Faecalicatena | 0.7100 | 0.0006 | 0.0162 |
|  | Eggerthella | 0.8500 | 0.0007 | 0.0162 |
|  | Propioniciclava | 0.7100 | 0.0005 | 0.0162 |
|  | Pseudactinotalea | 0.6700 | 0.0010 | 0.0169 |
|  | Georgenia | 0.6400 | 0.0008 | 0.0169 |
|  | Brachybacterium | 0.6500 | 0.0010 | 0.0169 |
|  | Flaviflexus | 0.6500 | 0.0008 | 0.0169 |
|  | Xanthomonas | 0.8500 | 0.0010 | 0.0169 |
|  | Actinotalea | 0.7700 | 0.0010 | 0.0169 |
|  | Miniimonas | 0.6800 | 0.0008 | 0.0169 |
|  | Peptacetobacter | 0.6900 | 0.0012 | 0.0179 |
|  | Cellulomonas | 0.6400 | 0.0012 | 0.0179 |
|  | Ruminiclostridium | 0.6800 | 0.0012 | 0.0179 |
|  | Tsukamurella | 0.7500 | 0.0014 | 0.0191 |
|  | Kutzneria | 0.7100 | 0.0014 | 0.0191 |
|  | Kineococcus | 0.7300 | 0.0017 | 0.0224 |
|  | Phascolarctobacterium | 0.6600 | 0.0021 | 0.0247 |
|  | Brevilactibacter | 0.6700 | 0.0022 | 0.0247 |
|  | Streptomyces | 0.5900 | 0.0025 | 0.0247 |
|  | Protaetiibacter | 0.6900 | 0.0025 | 0.0247 |
|  | Faecalibacterium | 0.5400 | 0.0023 | 0.0247 |
|  | Murdochiella | 0.6700 | 0.0024 | 0.0247 |
|  | Coprococcus | 0.6500 | 0.0024 | 0.0247 |
|  | Phycicoccus | 0.6400 | 0.0020 | 0.0247 |
|  | Massilistercora | 0.6200 | 0.0028 | 0.0268 |
|  | Actinotignum | 0.6500 | 0.0033 | 0.0297 |
|  | Ralstonia | 0.6900 | 0.0032 | 0.0297 |
|  | Mogibacterium | 0.5500 | 0.0033 | 0.0297 |
|  | Ornithinimicrobium | 0.6400 | 0.0035 | 0.0307 |
|  | Actinoplanes | 0.5700 | 0.0038 | 0.0312 |
|  | Pauljensenia | 0.5900 | 0.0038 | 0.0312 |
|  | Clostridium | 0.5000 | 0.0045 | 0.0356 |
|  | Actinobaculum | 0.5600 | 0.0045 | 0.0356 |
|  | Sorangium | 0.5200 | 0.0050 | 0.0364 |
|  | Rathayibacter | 0.6700 | 0.0049 | 0.0364 |
|  | Eubacterium | 0.6900 | 0.0050 | 0.0364 |
|  | Tessaracoccus | 0.5800 | 0.0049 | 0.0364 |
|  | Sinomonas | 0.6200 | 0.0052 | 0.0366 |
|  | Saccharothrix | 0.6300 | 0.0054 | 0.0370 |
|  | Clavibacter | 0.5600 | 0.0058 | 0.0370 |
|  | Lancefieldella | 0.5800 | 0.0055 | 0.0370 |
|  | Pseudobutyrivibrio | 0.5300 | 0.0059 | 0.0370 |
|  | Veillonella | 0.5200 | 0.0057 | 0.0370 |
|  | Arsenicicoccus | 0.5800 | 0.0056 | 0.0370 |
|  | Lachnospira | 0.5500 | 0.0064 | 0.0392 |
|  | Salmonella | 0.5900 | 0.0064 | 0.0392 |
|  | Pengzhenrongella | 0.5800 | 0.0065 | 0.0392 |
|  | Paraoerskovia | 0.6000 | 0.0068 | 0.0402 |
|  | Mageeibacillus | 0.5600 | 0.0070 | 0.0408 |
|  | Akkermansia | 0.5200 | 0.0076 | 0.0432 |
|  | Anaerobutyricum | 0.5200 | 0.0079 | 0.0439 |
|  | Paenibacillus | 0.4800 | 0.0079 | 0.0439 |
|  | Campylobacter | 0.5300 | 0.0081 | 0.0441 |
|  | Jonesia | 0.6000 | 0.0085 | 0.0444 |
|  | Ruthenibacterium | 0.5500 | 0.0085 | 0.0444 |
|  | Blautia | 0.5300 | 0.0084 | 0.0444 |
|  | Stenotrophomonas | 0.5700 | 0.0087 | 0.0446 |
|  | Nocardioides | 0.5800 | 0.0093 | 0.0468 |
|  | Xylanimonas | 0.5100 | 0.0094 | 0.0469 |
|  | Dermacoccus | 0.5900 | 0.0096 | 0.0470 |
|  | Hungatella | 0.5100 | 0.0104 | 0.0494 |
|  | Nanchangia | 0.5000 | 0.0106 | 0.0494 |
|  | Ruania | 0.5200 | 0.0106 | 0.0494 |
|  | Ruminococcus | 0.4700 | 0.0103 | 0.0494 |
|  | Bulleidia | 0.4600 | 0.0118 | 0.0539 |
|  | Acetivibrio | 0.5300 | 0.0120 | 0.0544 |
|  | Leifsonia | 0.5600 | 0.0124 | 0.0553 |
|  | Herbinix | 0.5400 | 0.0128 | 0.0553 |
|  | Brochothrix | 0.5200 | 0.0126 | 0.0553 |
|  | Dermabacter | 0.4900 | 0.0129 | 0.0553 |
|  | Actinomadura | 0.5200 | 0.0134 | 0.0563 |
|  | Changpingibacter | 0.5300 | 0.0133 | 0.0563 |
|  | Beutenbergia | 0.5700 | 0.0138 | 0.0571 |
|  | Mycolicibacterium | 0.5500 | 0.0147 | 0.0604 |
|  | Kitasatospora | 0.5700 | 0.0151 | 0.0604 |
|  | Buchnera | 0.4600 | 0.0150 | 0.0604 |
|  | Salinibacterium | 0.5300 | 0.0160 | 0.0632 |
|  | Janibacter | 0.5800 | 0.0164 | 0.0632 |
|  | Luteimicrobium | 0.5000 | 0.0163 | 0.0632 |
|  | Kytococcus | 0.5500 | 0.0167 | 0.0640 |
|  | Aerococcus | 0.4900 | 0.0183 | 0.0671 |
|  | Longicatena | 0.5500 | 0.0189 | 0.0671 |
|  | Rhodococcus | 0.5300 | 0.0186 | 0.0671 |
|  | Cellulosimicrobium | 0.5300 | 0.0178 | 0.0671 |
|  | Occultella | 0.4900 | 0.0187 | 0.0671 |
|  | Anaerostipes | 0.4700 | 0.0185 | 0.0671 |
|  | Cupriavidus | 0.5500 | 0.0188 | 0.0671 |
|  | Brevibacterium | 0.4600 | 0.0191 | 0.0671 |
|  | Propionibacterium | 0.6500 | 0.0193 | 0.0671 |
|  | Arachnia | 0.6500 | 0.0203 | 0.0699 |
|  | Trueperella | 0.4000 | 0.0223 | 0.0731 |
|  | Parvimonas | 0.6500 | 0.0215 | 0.0731 |
|  | Mobiluncus | 0.5200 | 0.0217 | 0.0731 |
|  | Enterocloster | 0.4600 | 0.0220 | 0.0731 |
|  | Collinsella | 0.4700 | 0.0221 | 0.0731 |
|  | Butyrivibrio | 0.4600 | 0.0229 | 0.0733 |
|  | Actinomyces | 0.4900 | 0.0226 | 0.0733 |
|  | Mycetocola | 0.5000 | 0.0228 | 0.0733 |
|  | Micrococcus | 0.4900 | 0.0243 | 0.0769 |
|  | Petrimonas | 0.4800 | 0.0246 | 0.0770 |
|  | Subtercola | 0.4500 | 0.0250 | 0.0778 |
|  | Dolosigranulum | 0.4400 | 0.0257 | 0.0785 |
|  | Agrobacterium | 0.5200 | 0.0255 | 0.0785 |
|  | Priestia | 0.4900 | 0.0265 | 0.0795 |
|  | Dialister | 0.5800 | 0.0264 | 0.0795 |
|  | Nakamurella | 0.4800 | 0.0278 | 0.0828 |
|  | Prevotella | 0.3900 | 0.0283 | 0.0836 |
|  | Acinetobacter | 0.4700 | 0.0295 | 0.0855 |
|  | Boudabousia | 0.5000 | 0.0294 | 0.0855 |
|  | Niallia | 0.4200 | 0.0304 | 0.0873 |
|  | Gemella | 0.5000 | 0.0312 | 0.0888 |
|  | Blastococcus | 0.5200 | 0.0316 | 0.0888 |
|  | Streptacidiphilus | 0.5000 | 0.0317 | 0.0888 |
|  | Streptococcus | 0.4600 | 0.0328 | 0.0905 |
|  | Selenomonas | 0.5100 | 0.0327 | 0.0905 |
|  | Aminipila | 0.5100 | 0.0335 | 0.0917 |
|  | Simonsiella | 0.6000 | 0.0346 | 0.0941 |
|  | Clostridioides | 0.3800 | 0.0352 | 0.0948 |
|  | Mammaliicoccus | 0.3500 | 0.0367 | 0.0982 |
|  | Dietzia | 0.4800 | 0.0386 | 0.0996 |
|  | Fastidiosipila | 0.4200 | 0.0381 | 0.0996 |
|  | Finegoldia | 0.3800 | 0.0389 | 0.0996 |
|  | Scardovia | 0.7700 | 0.0376 | 0.0996 |
|  | Metamycoplasma | 0.6800 | 0.0379 | 0.0996 |
|  | Amycolatopsis | 0.4800 | 0.0389 | 0.0996 |
| Species | *Sanguibacter sp. HDW7* | 0.8000 | 0.0000 | 0.0058 |
|  | *Anaerocolumna sedimenticola* | 0.8600 | 0.0000 | 0.0058 |
|  | *Cellulomonas sp. zg ZUI157* | 0.9400 | 0.0000 | 0.0058 |
|  | *Anaerostipes caccae* | 0.8200 | 0.0000 | 0.0066 |
|  | *Cellulomonas iranensis* | 0.8900 | 0.0000 | 0.0066 |
|  | *Sanguibacter keddieii* | 0.7500 | 0.0000 | 0.0067 |
|  | *Lachnoclostridium phocaeense* | 0.7900 | 0.0001 | 0.0129 |
|  | *Georgenia sp. Z443* | 0.6700 | 0.0001 | 0.0142 |
|  | *Brevibacterium casei* | 0.8700 | 0.0002 | 0.0160 |
|  | *Corynebacterium xerosis* | 0.8200 | 0.0002 | 0.0160 |
|  | *Microbacterium oxydans* | 0.9100 | 0.0003 | 0.0160 |
|  | *Blautia sp. NBRC 113351* | 0.7300 | 0.0003 | 0.0160 |
|  | *Erysipelatoclostridium ramosum* | 0.7100 | 0.0002 | 0.0160 |
|  | *Anaerotignum propionicum* | 0.7500 | 0.0003 | 0.0160 |
|  | *Blautia producta* | 0.7100 | 0.0002 | 0.0160 |
|  | *Moraxella osloensis* | 0.9600 | 0.0003 | 0.0160 |
|  | *Micropruina glycogenica* | 0.7900 | 0.0003 | 0.0160 |
|  | *Georgenia faecalis* | 0.6800 | 0.0004 | 0.0176 |
|  | *Oerskovia sp. KBS0722* | 0.7400 | 0.0004 | 0.0179 |
|  | *Petrocella atlantisensis* | 0.7300 | 0.0004 | 0.0187 |
|  | *Isoptericola variabilis* | 0.7600 | 0.0005 | 0.0193 |
|  | *Phascolarctobacterium sp. Marseille Q4147* | 0.7300 | 0.0006 | 0.0214 |
|  | *Anaerocolumna chitinilytica* | 0.6600 | 0.0006 | 0.0214 |
|  | *Propionibacterium australiense* | 0.7200 | 0.0006 | 0.0214 |
|  | *Cutibacterium avidum* | 0.6400 | 0.0006 | 0.0214 |
|  | *Brevilactibacter coleopterorum* | 0.6800 | 0.0007 | 0.0229 |
|  | *Anaerocolumna cellulosilytica* | 0.6600 | 0.0009 | 0.0233 |
|  | *Acidipropionibacterium jensenii* | 0.6300 | 0.0009 | 0.0233 |
|  | *Actinomyces respiraculi* | 0.5900 | 0.0008 | 0.0233 |
|  | *Butyrivibrio hungatei* | 0.6800 | 0.0010 | 0.0233 |
|  | *Lacrimispora sphenoides* | 0.6500 | 0.0010 | 0.0233 |
|  | *Faecalicatena sp. Marseille Q4148* | 0.6500 | 0.0010 | 0.0233 |
|  | *Rhodococcus fascians* | 0.7200 | 0.0009 | 0.0233 |
|  | *Leucobacter sp. CX169* | 0.8000 | 0.0009 | 0.0233 |
|  | *Cellulomonas sp. PSBB021* | 0.6800 | 0.0009 | 0.0233 |
|  | *X Clostridium scindens* | 0.6300 | 0.0010 | 0.0233 |
|  | *Miniimonas sp. S16* | 0.6400 | 0.0010 | 0.0233 |
|  | *Blautia hansenii* | 0.6600 | 0.0011 | 0.0233 |
|  | *Pseudactinotalea sp. HY158* | 0.6400 | 0.0011 | 0.0238 |
|  | *Brachybacterium sp. SGAir0954* | 0.6100 | 0.0012 | 0.0251 |
|  | *Streptomyces albus* | 0.7300 | 0.0012 | 0.0252 |
|  | *Actinotalea sp. JY 7876* | 0.7300 | 0.0013 | 0.0255 |
|  | *Brachybacterium saurashtrense* | 0.6800 | 0.0013 | 0.0255 |
|  | *Blautia sp. LZLJ 3* | 0.6400 | 0.0014 | 0.0269 |
|  | *Peptacetobacter hiranonis* | 0.6500 | 0.0015 | 0.0280 |
|  | *Actinomyces marmotae* | 0.5700 | 0.0017 | 0.0294 |
|  | *Rhizobium leguminosarum* | 0.7500 | 0.0016 | 0.0294 |
|  | *Brevibacterium aurantiacum* | 0.5900 | 0.0017 | 0.0294 |
|  | *Actinomyces slackii* | 0.5800 | 0.0017 | 0.0299 |
|  | *Actinomyces howellii* | 0.5600 | 0.0018 | 0.0300 |
|  | *Faecalibacterium prausnitzii* | 0.5100 | 0.0018 | 0.0304 |
|  | *Trueperella bialowiezensis* | 0.5900 | 0.0019 | 0.0310 |
|  | *Agrobacterium tumefaciens* | 0.7400 | 0.0020 | 0.0317 |
|  | *Actinomyces lilanjuaniae* | 0.5500 | 0.0021 | 0.0329 |
|  | *Actinomyces qiguomingii* | 0.5600 | 0.0023 | 0.0333 |
|  | *Ralstonia solanacearum* | 0.7000 | 0.0022 | 0.0333 |
|  | *Kineococcus radiotolerans* | 0.6900 | 0.0022 | 0.0333 |
|  | *Actinomyces sp. MAS 1* | 0.5600 | 0.0024 | 0.0344 |
|  | *Clostridium saccharoperbutylacetonicum* | 0.6000 | 0.0024 | 0.0344 |
|  | *Campylobacter curvus* | 0.6900 | 0.0026 | 0.0354 |
|  | *Corynebacterium frankenforstense* | 0.6700 | 0.0025 | 0.0354 |
|  | *Murdochiella vaginalis* | 0.6300 | 0.0027 | 0.0367 |
|  | *Georgenia sp. Z294* | 0.6100 | 0.0027 | 0.0367 |
|  | *Actinomyces sp. Chiba101* | 0.5500 | 0.0029 | 0.0370 |
|  | *Brevilactibacter sp. MC1595* | 0.6300 | 0.0029 | 0.0370 |
|  | *Dermabacter vaginalis* | 0.6000 | 0.0029 | 0.0370 |
|  | *Bifidobacterium thermophilum* | 0.6000 | 0.0029 | 0.0370 |
|  | *Paenibacillus polymyxa* | 0.7300 | 0.0030 | 0.0375 |
|  | *Isoptericola dokdonensis* | 0.6200 | 0.0031 | 0.0375 |
|  | *Cellulomonas taurus* | 0.5900 | 0.0031 | 0.0375 |
|  | *Ruminococcus sp. JE7A12* | 0.5500 | 0.0032 | 0.0378 |
|  | *Actinomyces sp. Z16* | 0.5300 | 0.0033 | 0.0387 |
|  | *Veillonella parvula* | 0.5500 | 0.0034 | 0.0392 |
|  | *Lachnoclostridium sp. YL32* | 0.7300 | 0.0035 | 0.0394 |
|  | *Gemella sp. zg 570* | 0.6000 | 0.0038 | 0.0394 |
|  | *Sorangium cellulosum* | 0.4900 | 0.0038 | 0.0394 |
|  | *Actinomyces procaprae* | 0.5200 | 0.0037 | 0.0394 |
|  | *Lachnoclostridium phytofermentans* | 0.5900 | 0.0037 | 0.0394 |
|  | *Cellulomonas sp. Y8* | 0.6100 | 0.0035 | 0.0394 |
|  | *Ruminiclostridium herbifermentans* | 0.6000 | 0.0036 | 0.0394 |
|  | *Actinobaculum sp. 313* | 0.5300 | 0.0036 | 0.0394 |
|  | *Actinomyces gaoshouyii* | 0.5200 | 0.0039 | 0.0397 |
|  | *Anaerostipes rhamnosivorans* | 0.5800 | 0.0040 | 0.0404 |
|  | *Bacillus thuringiensis* | 0.6400 | 0.0044 | 0.0412 |
|  | *Blautia argi* | 0.5500 | 0.0042 | 0.0412 |
|  | *Massilistercora timonensis* | 0.5600 | 0.0047 | 0.0412 |
|  | *Brachybacterium sp. P6 10 X1* | 0.6400 | 0.0043 | 0.0412 |
|  | *Corynebacterium striatum* | 0.4900 | 0.0045 | 0.0412 |
|  | *Cupriavidus basilensis* | 0.7200 | 0.0043 | 0.0412 |
|  | *Cellulomonas gilvus* | 0.6200 | 0.0045 | 0.0412 |
|  | *Corynebacterium choanae* | 0.6100 | 0.0047 | 0.0412 |
|  | *Actinotignum schaalii* | 0.6000 | 0.0047 | 0.0412 |
|  | *Veillonella dispar* | 0.4800 | 0.0046 | 0.0412 |
|  | *Clavibacter michiganensis* | 0.5500 | 0.0047 | 0.0412 |
|  | *Veillonella rodentium* | 0.4800 | 0.0044 | 0.0412 |
|  | *Acidipropionibacterium virtanenii* | 0.6000 | 0.0046 | 0.0412 |
|  | *Streptomyces lydicus* | 0.7500 | 0.0047 | 0.0412 |
|  | *Propionibacterium freudenreichii* | 0.5400 | 0.0051 | 0.0441 |
|  | *Streptococcus salivarius* | 0.6700 | 0.0053 | 0.0448 |
|  | *Lancefieldella parvula* | 0.5600 | 0.0054 | 0.0450 |
|  | *Rathayibacter sp. VKM Ac 2759* | 0.6800 | 0.0054 | 0.0450 |
|  | *Pauljensenia hongkongensis* | 0.5400 | 0.0058 | 0.0470 |
|  | *Blautia obeum* | 0.5300 | 0.0058 | 0.0470 |
|  | *Acinetobacter baumannii* | 0.6900 | 0.0057 | 0.0470 |
|  | *Coprococcus comes* | 0.5800 | 0.0061 | 0.0490 |
|  | *Aminipila terrae* | 0.6500 | 0.0062 | 0.0493 |
|  | *Acinetobacter johnsonii* | 0.7500 | 0.0063 | 0.0493 |
|  | *Arsenicicoccus sp. oral taxon 190* | 0.5600 | 0.0064 | 0.0493 |
|  | *Blautia pseudococcoides* | 0.5600 | 0.0063 | 0.0493 |
|  | *Actinomyces wuliandei* | 0.5000 | 0.0068 | 0.0497 |
|  | *Streptococcus rubneri* | 0.5000 | 0.0067 | 0.0497 |
|  | *Actinomyces faecalis* | 0.4700 | 0.0065 | 0.0497 |
|  | *Cellulomonas sp. zg ZUI188* | 0.5400 | 0.0067 | 0.0497 |
|  | *Pseudobutyrivibrio xylanivorans* | 0.4900 | 0.0068 | 0.0497 |
|  | *Streptococcus infantarius* | 0.5600 | 0.0068 | 0.0497 |
|  | *Salmonella enterica* | 0.5700 | 0.0068 | 0.0497 |
|  | *Microbacterium sp. WY121* | 0.6100 | 0.0069 | 0.0497 |
|  | *Cellulosimicrobium cellulans* | 0.6400 | 0.0070 | 0.0498 |
|  | *Actinomyces radicidentis* | 0.5000 | 0.0071 | 0.0503 |
|  | *Bifidobacterium choerinum* | 0.6100 | 0.0072 | 0.0503 |
|  | *Enterococcus avium* | 0.4800 | 0.0075 | 0.0515 |
|  | *Stenotrophomonas maltophilia* | 0.5900 | 0.0074 | 0.0515 |
|  | *Streptococcus sp. A12* | 0.5300 | 0.0079 | 0.0518 |
|  | *Streptococcus koreensis* | 0.5200 | 0.0078 | 0.0518 |
|  | *Streptococcus ilei* | 0.5200 | 0.0077 | 0.0518 |
|  | *Vagococcus zengguangii* | 0.5500 | 0.0080 | 0.0518 |
|  | *Janibacter limosus* | 0.6400 | 0.0081 | 0.0518 |
|  | *Humibacter sp. WJ7 1* | 0.5900 | 0.0082 | 0.0518 |
|  | *Bacteroides nordii* | 0.5400 | 0.0080 | 0.0518 |
|  | *Pengzhenrongella sicca* | 0.5400 | 0.0079 | 0.0518 |
|  | *Streptococcus iniae* | 0.4600 | 0.0083 | 0.0518 |
|  | *Butyrivibrio proteoclasticus* | 0.5300 | 0.0076 | 0.0518 |
|  | *Streptococcus canis* | 0.4600 | 0.0083 | 0.0518 |
|  | *Lachnospira eligens* | 0.5100 | 0.0083 | 0.0518 |
|  | *Clostridium sp. SY8519* | 0.5400 | 0.0080 | 0.0518 |
|  | *Microbacterium protaetiae* | 0.6000 | 0.0084 | 0.0518 |
|  | *Cellulomonas sp. H30R 01* | 0.5700 | 0.0084 | 0.0518 |
|  | *X Ruminococcus torques* | 0.5000 | 0.0085 | 0.0519 |
|  | *Paraoerskovia marina* | 0.5700 | 0.0086 | 0.0524 |
|  | *Corynebacterium pelargi* | 0.5900 | 0.0088 | 0.0533 |
|  | *Actinomyces sp. 432* | 0.4800 | 0.0089 | 0.0533 |
|  | *Schaalia sp. 19OD2882* | 0.4600 | 0.0091 | 0.0539 |
|  | *Streptococcus halotolerans* | 0.4400 | 0.0096 | 0.0541 |
|  | *Actinomyces weissii* | 0.4800 | 0.0094 | 0.0541 |
|  | *Veillonella sp. S12025 13* | 0.4400 | 0.0095 | 0.0541 |
|  | *Clostridium sp. CT4* | 0.5200 | 0.0093 | 0.0541 |
|  | *Mageeibacillus indolicus* | 0.5100 | 0.0096 | 0.0541 |
|  | *Corynebacterium tuberculostearicum* | 0.5500 | 0.0095 | 0.0541 |
|  | *Fusobacterium gonidiaformans* | 0.5500 | 0.0096 | 0.0541 |
|  | *Anaerobutyricum hallii* | 0.4800 | 0.0098 | 0.0544 |
|  | *Acidipropionibacterium acidipropionici* | 0.5500 | 0.0097 | 0.0544 |
|  | *Phycicoccus sp. HDW14* | 0.5200 | 0.0098 | 0.0544 |
|  | *Clostridium butyricum* | 0.4900 | 0.0100 | 0.0549 |
|  | *Ruania sp. HY168* | 0.5700 | 0.0100 | 0.0549 |
|  | *Corynebacterium falsenii* | 0.5300 | 0.0106 | 0.0576 |
|  | *Brachybacterium avium* | 0.5400 | 0.0108 | 0.0577 |
|  | *Arthrobacter agilis* | 0.4900 | 0.0108 | 0.0577 |
|  | *Sinomonas atrocyanea* | 0.5500 | 0.0107 | 0.0577 |
|  | *Flaviflexus ciconiae* | 0.5000 | 0.0109 | 0.0580 |
|  | *Rhodopseudomonas palustris* | 0.5500 | 0.0113 | 0.0597 |
|  | *Nanchangia anserum* | 0.4800 | 0.0115 | 0.0598 |
|  | *Streptomyces venezuelae* | 0.5600 | 0.0114 | 0.0598 |
|  | *Bifidobacterium pseudolongum* | 0.4700 | 0.0116 | 0.0598 |
|  | *Priestia megaterium* | 0.5600 | 0.0119 | 0.0599 |
|  | *Bacteroides helcogenes* | 0.4900 | 0.0117 | 0.0599 |
|  | *Jonesia denitrificans* | 0.5500 | 0.0119 | 0.0599 |
|  | *Actinomyces israelii* | 0.4600 | 0.0117 | 0.0599 |
|  | *Bulleidia sp. zg 1006* | 0.4300 | 0.0119 | 0.0599 |
|  | *Schaalia meyeri* | 0.4600 | 0.0120 | 0.0600 |
|  | *Dermacoccus nishinomiyaensis* | 0.5600 | 0.0124 | 0.0613 |
|  | *Flaviflexus salsibiostraticola* | 0.4700 | 0.0125 | 0.0618 |
|  | *Arachnia propionica* | 0.6800 | 0.0127 | 0.0621 |
|  | *Blautia massiliensis* | 0.6200 | 0.0129 | 0.0627 |
|  | *Campylobacter gracilis* | 0.6900 | 0.0134 | 0.0648 |
|  | *Flaviflexus sp. dk850* | 0.4700 | 0.0136 | 0.0657 |
|  | *Eubacterium sp. NSJ 61* | 0.5400 | 0.0140 | 0.0664 |
|  | *Pseudomonas fluorescens* | 0.5700 | 0.0139 | 0.0664 |
|  | *Ruthenibacterium lactatiformans* | 0.4900 | 0.0140 | 0.0664 |
|  | *Saccharopolyspora erythraea* | 0.5600 | 0.0142 | 0.0667 |
|  | *Streptococcus thermophilus* | 0.5300 | 0.0144 | 0.0673 |
|  | *Tsukamurella paurometabola* | 0.5700 | 0.0144 | 0.0673 |
|  | *Tessaracoccus flavescens* | 0.5600 | 0.0146 | 0.0677 |
|  | *Clostridium beijerinckii* | 0.5400 | 0.0148 | 0.0682 |
|  | *Corynebacterium urealyticum* | 0.5000 | 0.0149 | 0.0683 |
|  | *Cellulomonas shaoxiangyii* | 0.4700 | 0.0152 | 0.0684 |
|  | *Buchnera aphidicola* | 0.4300 | 0.0151 | 0.0684 |
|  | *Cryobacterium sp. LW097* | 0.5400 | 0.0152 | 0.0684 |
|  | *Corynebacterium lizhenjunii* | 0.5300 | 0.0154 | 0.0686 |
|  | *Bacteroides thetaiotaomicron* | 0.4000 | 0.0154 | 0.0686 |
|  | *Leucobacter triazinivorans* | 0.5200 | 0.0155 | 0.0687 |
|  | *Herbinix luporum* | 0.4900 | 0.0158 | 0.0696 |
|  | *Corynebacterium maris* | 0.5200 | 0.0160 | 0.0696 |
|  | *Streptococcus australis* | 0.4700 | 0.0160 | 0.0696 |
|  | *Arcanobacterium sp. JY X040* | 0.4900 | 0.0160 | 0.0696 |
|  | *Ruania alkalisoli* | 0.5000 | 0.0162 | 0.0699 |
|  | *Veillonella nakazawae* | 0.4100 | 0.0163 | 0.0700 |
|  | *Bifidobacterium asteroides* | 0.5100 | 0.0165 | 0.0707 |
|  | *Hungatella hathewayi* | 0.4600 | 0.0168 | 0.0710 |
|  | *Streptococcus halichoeri* | 0.4400 | 0.0168 | 0.0710 |
|  | *X Ruminococcus gnavus* | 0.4800 | 0.0168 | 0.0710 |
|  | *Dermacoccus sp. PAMC28757* | 0.5200 | 0.0170 | 0.0714 |
|  | *Phascolarctobacterium faecium* | 0.4800 | 0.0171 | 0.0715 |
|  | *Clostridium pasteurianum* | 0.4500 | 0.0172 | 0.0715 |
|  | *Enterococcus faecium* | 0.4600 | 0.0174 | 0.0719 |
|  | *Mammaliicoccus sciuri* | 0.3900 | 0.0176 | 0.0719 |
|  | *Selenomonas timonae* | 0.5600 | 0.0175 | 0.0719 |
|  | *Dolosigranulum pigrum* | 0.4300 | 0.0186 | 0.0754 |
|  | *Dermabacter jinjuensis* | 0.4300 | 0.0187 | 0.0754 |
|  | *Rhodococcus opacus* | 0.6000 | 0.0187 | 0.0754 |
|  | *Beutenbergia cavernae* | 0.5200 | 0.0189 | 0.0755 |
|  | *Enterocloster bolteae* | 0.4400 | 0.0192 | 0.0755 |
|  | *Streptococcus sp. FDAARGOS_192* | 0.5800 | 0.0191 | 0.0755 |
|  | *Xylanimonas allomyrinae* | 0.4800 | 0.0193 | 0.0755 |
|  | *Corynebacterium imitans* | 0.4900 | 0.0193 | 0.0755 |
|  | *Salinibacterium sp. UTAS2018* | 0.5600 | 0.0193 | 0.0755 |
|  | *Bifidobacterium animalis* | 0.5300 | 0.0193 | 0.0755 |
|  | *Campylobacter concisus* | 0.4700 | 0.0195 | 0.0757 |
|  | *Gemella morbillorum* | 0.5300 | 0.0198 | 0.0757 |
|  | *Collinsella aerofaciens* | 0.4600 | 0.0196 | 0.0757 |
|  | *Mogibacterium diversum* | 0.5200 | 0.0198 | 0.0757 |
|  | *Brachybacterium faecium* | 0.5200 | 0.0198 | 0.0757 |
|  | *Nocardia brasiliensis* | 0.5200 | 0.0201 | 0.0758 |
|  | *Bacteroides intestinalis* | 0.4900 | 0.0201 | 0.0758 |
|  | *Coprococcus catus* | 0.4500 | 0.0200 | 0.0758 |
|  | *Bacillus cereus* | 0.4800 | 0.0205 | 0.0766 |
|  | *Bifidobacterium pseudocatenulatum* | 0.5600 | 0.0205 | 0.0766 |
|  | *Brochothrix thermosphacta* | 0.4700 | 0.0213 | 0.0792 |
|  | *Selenomonas sp. oral taxon 920* | 0.5400 | 0.0215 | 0.0793 |
|  | *Kytococcus sedentarius* | 0.5100 | 0.0215 | 0.0793 |
|  | *Luteimicrobium xylanilyticum* | 0.4600 | 0.0218 | 0.0799 |
|  | *Arthrobacter woluwensis* | 0.4800 | 0.0219 | 0.0799 |
|  | *Ruania sp. HY164* | 0.4600 | 0.0221 | 0.0803 |
|  | *Streptococcus sp. DAT741* | 0.4800 | 0.0226 | 0.0818 |
|  | *Xylanimicrobium sp. FW10M 9* | 0.5000 | 0.0228 | 0.0822 |
|  | *Megamonas hypermegale* | 0.4500 | 0.0232 | 0.0827 |
|  | *Akkermansia muciniphila* | 0.4300 | 0.0231 | 0.0827 |
|  | *Streptococcus sp. HSISS1* | 0.6400 | 0.0235 | 0.0834 |
|  | *Microlunatus sagamiharensis* | 0.5400 | 0.0236 | 0.0835 |
|  | *Bifidobacterium actinocoloniiforme* | 0.5600 | 0.0238 | 0.0839 |
|  | *Clostridium baratii* | 0.4600 | 0.0244 | 0.0856 |
|  | *Arthrobacter sp. 24S4 2* | 0.5000 | 0.0245 | 0.0857 |
|  | *Arthrobacter sp. PM3* | 0.5100 | 0.0251 | 0.0873 |
|  | *Corynebacterium aquilae* | 0.4700 | 0.0255 | 0.0885 |
|  | *Mobiluncus curtisii* | 0.4800 | 0.0257 | 0.0887 |
|  | *Streptococcus respiraculi* | 0.3800 | 0.0261 | 0.0898 |
|  | *Aminipila butyrica* | 0.5400 | 0.0266 | 0.0909 |
|  | *Porphyromonas asaccharolytica* | 0.5100 | 0.0266 | 0.0909 |
|  | *Streptococcus parauberis* | 0.3800 | 0.0267 | 0.0909 |
|  | *Trueperella pecoris* | 0.3900 | 0.0270 | 0.0913 |
|  | *Parvimonas micra* | 0.6000 | 0.0271 | 0.0914 |
|  | *Clostridium sp. C1* | 0.4800 | 0.0276 | 0.0925 |
|  | *Clostridium botulinum* | 0.5200 | 0.0279 | 0.0927 |
|  | *Streptococcus equinus* | 0.3800 | 0.0280 | 0.0927 |
|  | *Streptomyces cattleya* | 0.5400 | 0.0279 | 0.0927 |
|  | *Selenomonas sp. oral taxon 126* | 0.5200 | 0.0280 | 0.0927 |
|  | *Tessaracoccus timonensis* | 0.4800 | 0.0282 | 0.0929 |
|  | *Leptotrichia sp. oral taxon 847* | 0.5600 | 0.0283 | 0.0930 |
|  | *Blautia sp. SC05B48* | 0.5100 | 0.0293 | 0.0957 |
|  | *Streptococcus sp. zg 86* | 0.3700 | 0.0294 | 0.0957 |
|  | *Intrasporangium calvum* | 0.4900 | 0.0295 | 0.0958 |
|  | *Cellulomonas fimi* | 0.5100 | 0.0301 | 0.0971 |
|  | *Tessaracoccus sp. J1M15* | 0.5000 | 0.0304 | 0.0978 |
|  | *Gemella sp. oral taxon 928* | 0.4200 | 0.0307 | 0.0983 |
|  | *Anaerostipes hadrus* | 0.4300 | 0.0308 | 0.0984 |
|  | *Longicatena caecimuris* | 0.5000 | 0.0312 | 0.0990 |
|  | *Selenomonas sputigena* | 0.6000 | 0.0311 | 0.0990 |
|  | *Butyrivibrio fibrisolvens* | 0.4100 | 0.0318 | 0.0997 |
|  | *Streptococcus marmotae* | 0.4100 | 0.0317 | 0.0997 |
|  | *Dialister pneumosintes* | 0.5600 | 0.0319 | 0.0997 |
|  | *Microbacterium sp. XT11* | 0.4700 | 0.0319 | 0.0997 |

**Legend to Table 5:** Linear contrasts have been constructed and tested for significance using voom:limma approach combined with GMPR normalization. Adjustment for multiple testing was done using Benjamini-Hochberg false discovery rate withing each taxonomic level. Reported are findings with FDR <10% in at least one contrasts. No significant results were obtained for comparisons between baseline and six weeks

**Supplementary Table 6:** Differentially abundant taxa in saliva microbiota between RA patients who achieved minimum clinically important improvement (MCII+) at baseline and six weeks treatment

| **Taxonomic level** | **Taxa** | **MCII+ between baseline and six weeks** | | |
| --- | --- | --- | --- | --- |
|  |  | **logFC** | **P.Value.mcpos** | **P adj val** |
| **Species** | *Staphylococcus aureus* | 0·930 | 0·001 | 0·029 |
|  | *Actinomyces weissii* | 0·940 | 0·001 | 0·029 |
|  | *Actinomyces respiraculi* | 0·950 | 0·001 | 0·029 |
|  | *Muribaculum intestinale* | 0·960 | 0·001 | 0·029 |
|  | *Schaalia meyeri* | 0·960 | 0·001 | 0·029 |
|  | *Actinomyces sp 432* | 0·980 | 0·001 | 0·029 |
|  | *Actinomyces lilanjuaniae* | 0·990 | 0·000 | 0·029 |
|  | *Actinomyces procaprae* | 0·990 | 0·001 | 0·029 |
|  | *Actinomyces wuliandei* | 0·990 | 0·001 | 0·029 |
|  | *Actinomyces sp Chiba101* | 1·000 | 0·001 | 0·029 |
|  | *Actinomyces howellii* | 1·010 | 0·000 | 0·029 |
|  | *Actinomyces marmotae* | 1·010 | 0·000 | 0·029 |
|  | *Actinomyces slackii* | 1·020 | 0·001 | 0·029 |
|  | *Actinomyces gaoshouyii* | 1·030 | 0·000 | 0·029 |
|  | *Pauljensenia hongkongensis* | 1·050 | 0·001 | 0·029 |
|  | *Actinomyces sp MAS 1* | 1·060 | 0·000 | 0·029 |
|  | *Gemella sanguinis* | 1·110 | 0·001 | 0·029 |
|  | *Mogibacterium diversum* | 1·110 | 0·001 | 0·029 |
|  | *Actinomyces viscosus* | 1·210 | 0·001 | 0·029 |
|  | *Beutenbergia cavernae* | 1·230 | 0·000 | 0·029 |
|  | *Leptotrichia trevisanii* | 1·240 | 0·001 | 0·029 |
|  | *Leptotrichia hofstadii* | 1·340 | 0·001 | 0·029 |
|  | *Leptotrichia hongkongensis* | 1·590 | 0·000 | 0·029 |
|  | *Kingella oralis* | 1·400 | 0·001 | 0·031 |
|  | *Faecalibacterium prausnitzii* | 0·810 | 0·001 | 0·033 |
|  | *Arachnia propionica* | 1·350 | 0·001 | 0·033 |
|  | *Streptococcus sp oral taxon 061* | 0·930 | 0·002 | 0·035 |
|  | *Clavibacter michiganensis* | 1·000 | 0·001 | 0·035 |
|  | *Actinomyces sp HMT 175* | 1·170 | 0·002 | 0·035 |
|  | *Actinomyces sp Z16* | 0·900 | 0·002 | 0·035 |
|  | *Gemella morbillorum* | 1·090 | 0·002 | 0·038 |
|  | *Leptotrichia sp oral taxon 847* | 1·120 | 0·002 | 0·038 |
|  | *Prevotella copri* | 0·760 | 0·002 | 0·038 |
|  | *Flaviflexus sp dk850* | 0·830 | 0·002 | 0·038 |
|  | *Sanguibacter sp HDW7* | 0·860 | 0·002 | 0·038 |
|  | *Schaalia sp 19OD2882* | 0·860 | 0·002 | 0·038 |
|  | *Actinomyces qiguomingii* | 0·910 | 0·002 | 0·038 |
|  | *Streptococcus sanguinis* | 1·080 | 0·002 | 0·038 |
|  | *Actinomyces oris* | 1·090 | 0·002 | 0·038 |
|  | *Actinomyces israelii* | 0·870 | 0·003 | 0·042 |
|  | *Actinomyces radicidentis* | 0·940 | 0·003 | 0·042 |
|  | *Veillonella rodentium* | 0·770 | 0·003 | 0·042 |
|  | *Actinomyces faecalis* | 0·820 | 0·003 | 0·042 |
|  | *Actinomyces trachealis* | 0·830 | 0·003 | 0·042 |
|  | *Streptococcus ferus* | 0·850 | 0·003 | 0·042 |
|  | *Cellulomonas sp zg ZUI157* | 0·970 | 0·003 | 0·042 |
|  | *Eikenella corrodens* | 1·020 | 0·003 | 0·042 |
|  | *Leptotrichia sp oral taxon 498* | 1·170 | 0·003 | 0·042 |
|  | *Leptotrichia buccalis* | 1·180 | 0·003 | 0·042 |
|  | *Parvimonas micra* | 1·220 | 0·003 | 0·042 |
|  | *Campylobacter rectus* | 1·320 | 0·003 | 0·042 |
|  | *Campylobacter concisus* | 0·880 | 0·003 | 0·042 |
|  | *Corynebacterium maris* | 0·950 | 0·004 | 0·043 |
|  | *Capnocytophaga endodontalis* | 1·120 | 0·004 | 0·043 |
|  | *Streptococcus rubneri* | 0·870 | 0·004 | 0·043 |
|  | *Actinomyces naeslundii* | 1·090 | 0·004 | 0·043 |
|  | *Streptococcus iniae* | 0·810 | 0·004 | 0·043 |
|  | *Streptococcus sp oral taxon 431* | 0·920 | 0·004 | 0·043 |
|  | *Streptococcus gordonii* | 0·990 | 0·004 | 0·043 |
|  | *Actinomyces sp oral taxon 169* | 1·120 | 0·004 | 0·043 |
|  | *Prevotella sp oral taxon 475* | 0·780 | 0·004 | 0·044 |
|  | *Riemerella anatipestifer* | 0·850 | 0·004 | 0·044 |
|  | *Streptococcus uberis* | 0·850 | 0·004 | 0·044 |
|  | *Streptococcus agalactiae* | 0·910 | 0·004 | 0·044 |
|  | *Actinomyces sp oral taxon 171* | 1·080 | 0·004 | 0·044 |
|  | *Streptococcus halotolerans* | 0·760 | 0·005 | 0·048 |
|  | *Streptococcus pseudoporcinus* | 0·760 | 0·005 | 0·048 |
|  | *Herbinix luporum* | 0·790 | 0·006 | 0·048 |
|  | *Streptococcus himalayensis* | 0·790 | 0·005 | 0·048 |
|  | *Corynebacterium gerontici* | 0·810 | 0·006 | 0·048 |
|  | *Enterococcus faecium* | 0·830 | 0·005 | 0·048 |
|  | *Streptococcus australis* | 0·850 | 0·005 | 0·048 |
|  | *Streptococcus periodonticum* | 0·950 | 0·005 | 0·048 |
|  | *Candidatus Saccharimonas aalborgensis* | 1·020 | 0·006 | 0·048 |
|  | *Campylobacter showae* | 1·040 | 0·006 | 0·048 |
|  | *Actinomyces sp oral taxon 414* | 1·070 | 0·006 | 0·048 |
|  | *Fusobacterium sp oral taxon 203* | 1·160 | 0·005 | 0·048 |
|  | *Campylobacter gracilis* | 1·180 | 0·006 | 0·048 |
|  | *Schaalia odontolytica* | 0·740 | 0·006 | 0·050 |
|  | *Clostridioides difficile* | 0·710 | 0·006 | 0·051 |
|  | *Georgenia sp Z443* | 0·740 | 0·006 | 0·051 |
|  | *Streptococcus sp A12* | 0·870 | 0·006 | 0·051 |
|  | *Capnocytophaga sputigena* | 1·030 | 0·007 | 0·052 |
|  | *Schaalia turicensis* | 0·770 | 0·007 | 0·054 |
|  | *Bacteroides stercoris* | 0·780 | 0·007 | 0·054 |
|  | *Corynebacterium ulcerans* | 0·880 | 0·007 | 0·054 |
|  | *Eikenella exigua* | 0·940 | 0·007 | 0·054 |
|  | *Streptococcus sp 116 D4* | 0·900 | 0·008 | 0·057 |
|  | *Streptococcus koreensis* | 0·840 | 0·008 | 0·057 |
|  | *Streptococcus porcinus* | 0·700 | 0·009 | 0·058 |
|  | *Streptococcus parauberis* | 0·740 | 0·008 | 0·058 |
|  | *Fusobacterium necrophorum* | 0·870 | 0·009 | 0·058 |
|  | *Campylobacter curvus* | 0·900 | 0·008 | 0·058 |
|  | *Leptotrichia sp oral taxon 218* | 0·910 | 0·009 | 0·058 |
|  | *Moraxella osloensis* | 1·020 | 0·009 | 0·058 |
|  | *Tannerella sp oral taxon HOT 286* | 1·100 | 0·009 | 0·058 |
|  | *Treponema socranskii* | 1·120 | 0·008 | 0·058 |
|  | *Mycoplasma salivarium* | 1·420 | 0·009 | 0·058 |
|  | *Prevotella sp Rep29* | 0·680 | 0·010 | 0·059 |
|  | *Sodaliphilus pleomorphus* | 0·700 | 0·009 | 0·059 |
|  | *Georgenia faecalis* | 0·720 | 0·010 | 0·059 |
|  | *Streptococcus gallolyticus* | 0·720 | 0·009 | 0·059 |
|  | *Streptococcus pluranimalium* | 0·730 | 0·009 | 0·059 |
|  | *Pseudactinotalea sp HY158* | 0·740 | 0·009 | 0·059 |
|  | *Fusobacterium hwasookii* | 0·800 | 0·009 | 0·059 |
|  | *Streptococcus anginosus* | 0·810 | 0·010 | 0·059 |
|  | *Streptococcus cristatus* | 0·970 | 0·009 | 0·059 |
|  | *Dialister pneumosintes* | 1·050 | 0·010 | 0·059 |
|  | *Pseudoprevotella muciniphila* | 0·730 | 0·010 | 0·059 |
|  | *Prevotella nigrescens* | 0·740 | 0·010 | 0·059 |
|  | *Streptococcus equi* | 0·660 | 0·010 | 0·059 |
|  | *Streptococcus sp 1643* | 0·870 | 0·010 | 0·060 |
|  | *Dolosigranulum pigrum* | 0·730 | 0·011 | 0·061 |
|  | *Tannerella forsythia* | 1·160 | 0·011 | 0·061 |
|  | *Streptococcus equinus* | 0·710 | 0·011 | 0·062 |
|  | *Veillonella dispar* | 0·670 | 0·011 | 0·063 |
|  | *Veillonella parvula* | 0·710 | 0·011 | 0·063 |
|  | *Treponema parvum* | 1·070 | 0·011 | 0·063 |
|  | *Trueperella pecoris* | 0·670 | 0·012 | 0·063 |
|  | *Anaerotignum propionicum* | 0·710 | 0·012 | 0·063 |
|  | *Schaalia sp ZJ405* | 0·770 | 0·012 | 0·063 |
|  | *Prevotella sp oral taxon 299* | 0·830 | 0·012 | 0·063 |
|  | *Pseudoleptotrichia goodfellowii* | 0·940 | 0·012 | 0·063 |
|  | *Actinobaculum sp 313* | 0·700 | 0·012 | 0·064 |
|  | *Streptococcus sp HKU75* | 0·710 | 0·012 | 0·064 |
|  | *Streptococcus ilei* | 0·790 | 0·012 | 0·064 |
|  | *Streptococcus sp NPS 308* | 0·860 | 0·013 | 0·065 |
|  | *Mogibacterium pumilum* | 0·700 | 0·013 | 0·065 |
|  | *Alistipes onderdonkii* | 0·840 | 0·013 | 0·065 |
|  | *Capnocytophaga haemolytica* | 0·900 | 0·013 | 0·065 |
|  | *Streptococcus sp zg 86* | 0·700 | 0·013 | 0·066 |
|  | *Candidatus Nanosynbacter lyticus* | 1·000 | 0·013 | 0·066 |
|  | *Neisseria sp KEM232* | 1·020 | 0·014 | 0·066 |
|  | *Corynebacterium matruchotii* | 1·380 | 0·013 | 0·066 |
|  | *Neisseria lactamica* | 1·060 | 0·014 | 0·066 |
|  | *Sorangium cellulosum* | 0·630 | 0·014 | 0·068 |
|  | *Streptococcus canis* | 0·660 | 0·015 | 0·068 |
|  | *Streptococcus respiraculi* | 0·660 | 0·015 | 0·068 |
|  | *Streptococcus dysgalactiae* | 0·690 | 0·015 | 0·068 |
|  | *Granulicatella adiacens* | 0·720 | 0·015 | 0·068 |
|  | *Streptococcus suis* | 0·720 | 0·014 | 0·068 |
|  | *Prevotella melaninogenica* | 0·770 | 0·015 | 0·068 |
|  | *Ralstonia solanacearum* | 0·810 | 0·015 | 0·068 |
|  | *Capnocytophaga sp oral taxon 878* | 0·870 | 0·015 | 0·068 |
|  | *Capnocytophaga gingivalis* | 0·970 | 0·015 | 0·068 |
|  | *Arachnia rubra* | 1·130 | 0·015 | 0·068 |
|  | *Desulfobulbus oralis* | 1·240 | 0·015 | 0·068 |
|  | *Prevotella sp WR041* | 0·640 | 0·016 | 0·068 |
|  | *Treponema sp Marseille Q4132* | 1·030 | 0·016 | 0·068 |
|  | *Cardiobacterium hominis* | 1·220 | 0·016 | 0·068 |
|  | *Lachnoanaerobaculum umeaense* | 0·610 | 0·017 | 0·069 |
|  | *Veillonella sp S12025 13* | 0·610 | 0·017 | 0·069 |
|  | *Prevotella fusca* | 0·620 | 0·016 | 0·069 |
|  | *Corynebacterium aquilae* | 0·710 | 0·016 | 0·069 |
|  | *Prevotella enoeca* | 0·800 | 0·017 | 0·069 |
|  | *Selenomonas timonae* | 0·860 | 0·016 | 0·069 |
|  | *Porphyromonas sp oral taxon 275* | 0·980 | 0·016 | 0·069 |
|  | *Cardiobacterium sp Marseille Q4385* | 1·120 | 0·016 | 0·069 |
|  | *Corynebacterium testudinoris* | 0·660 | 0·017 | 0·069 |
|  | *Trueperella pyogenes* | 0·690 | 0·017 | 0·069 |
|  | *Brevibacterium casei* | 0·900 | 0·017 | 0·069 |
|  | *Pengzhenrongella sicca* | 0·700 | 0·017 | 0·070 |
|  | *Selenomonas sputigena* | 0·980 | 0·018 | 0·070 |
|  | *Fusobacterium canifelinum* | 0·790 | 0·018 | 0·070 |
|  | *Phocaeicola salanitronis* | 0·580 | 0·018 | 0·070 |
|  | *Fusobacterium nucleatum* | 0·810 | 0·018 | 0·071 |
|  | *Peptacetobacter hiranonis* | 0·730 | 0·018 | 0·072 |
|  | *Fretibacterium fastidiosum* | 1·180 | 0·019 | 0·075 |
|  | *Enterococcus avium* | 0·670 | 0·020 | 0·076 |
|  | *Neisseria musculi* | 0·950 | 0·020 | 0·076 |
|  | *Streptococcus pseudopneumoniae* | 0·850 | 0·020 | 0·076 |
|  | *Prevotella buccalis* | 0·610 | 0·020 | 0·076 |
|  | *Prevotella intermedia* | 0·610 | 0·020 | 0·077 |
|  | *Muribaculum gordoncarteri* | 0·640 | 0·020 | 0·077 |
|  | *Capnocytophaga sp oral taxon 323* | 0·890 | 0·021 | 0·077 |
|  | *Leptotrichia shahii* | 0·830 | 0·021 | 0·077 |
|  | *Blautia sp NBRC 113351* | 0·670 | 0·021 | 0·078 |
|  | *Neisseria flavescens* | 1·090 | 0·021 | 0·078 |
|  | *Parabacteroides goldsteinii* | 0·640 | 0·022 | 0·078 |
|  | *Neisseria meningitidis* | 1·060 | 0·022 | 0·078 |
|  | *Streptomyces venezuelae* | 0·810 | 0·022 | 0·079 |
|  | *Selenomonas sp oral taxon 126* | 0·840 | 0·022 | 0·079 |
|  | *Streptococcus pneumoniae* | 0·790 | 0·022 | 0·080 |
|  | *Neisseria chenwenguii* | 0·860 | 0·023 | 0·080 |
|  | *Neisseria gonorrhoeae* | 1·050 | 0·023 | 0·080 |
|  | *Finegoldia magna* | 0·590 | 0·023 | 0·081 |
|  | *Corynebacterium striatum* | 0·630 | 0·024 | 0·081 |
|  | *Streptococcus parasuis* | 0·650 | 0·024 | 0·081 |
|  | *Bacillus cereus* | 0·720 | 0·024 | 0·081 |
|  | *Brevibacterium luteolum* | 0·750 | 0·024 | 0·081 |
|  | *Capnocytophaga sp oral taxon 864* | 0·830 | 0·024 | 0·081 |
|  | *Aggregatibacter aphrophilus* | 0·970 | 0·024 | 0·081 |
|  | *Prevotella ruminicola* | 0·560 | 0·024 | 0·081 |
|  | *Anaerobutyricum hallii* | 0·630 | 0·024 | 0·081 |
|  | *Streptococcus intermedius* | 0·710 | 0·025 | 0·081 |
|  | *Prevotella oris* | 0·770 | 0·025 | 0·081 |
|  | *Rothia aeria* | 0·800 | 0·025 | 0·081 |
|  | *Cutibacterium acnes* | 0·850 | 0·025 | 0·081 |
|  | *Gemella sp zg 570* | 0·720 | 0·025 | 0·081 |
|  | *Leptotrichia sp oral taxon 212* | 0·810 | 0·025 | 0·081 |
|  | *Anaerocolumna sedimenticola* | 0·630 | 0·025 | 0·082 |
|  | *Flaviflexus ciconiae* | 0·680 | 0·025 | 0·082 |
|  | *Neisseria canis* | 0·820 | 0·026 | 0·082 |
|  | *Streptococcus sp KS 6* | 0·830 | 0·025 | 0·082 |
|  | *Prevotella veroralis* | 0·840 | 0·026 | 0·083 |
|  | *Nanchangia anserum* | 0·620 | 0·026 | 0·083 |
|  | *Alistipes megaguti* | 0·680 | 0·026 | 0·083 |
|  | *Capnocytophaga sp oral taxon 902* | 0·820 | 0·027 | 0·084 |
|  | *Prevotella scopos* | 0·630 | 0·027 | 0·084 |
|  | *Streptococcus ratti* | 0·630 | 0·027 | 0·084 |
|  | *Helcococcus kunzii* | 0·690 | 0·027 | 0·084 |
|  | *Corynebacterium xerosis* | 0·700 | 0·027 | 0·084 |
|  | *Olsenella uli* | 0·880 | 0·028 | 0·085 |
|  | *Capnocytophaga ochracea* | 0·860 | 0·029 | 0·086 |
|  | *Leptotrichia wadei* | 0·860 | 0·028 | 0·086 |
|  | *Neisseria zalophi* | 0·900 | 0·029 | 0·086 |
|  | *Streptococcus halichoeri* | 0·630 | 0·029 | 0·086 |
|  | *Pseudomonas putida* | 0·740 | 0·029 | 0·086 |
|  | *Streptococcus merionis* | 0·620 | 0·029 | 0·087 |
|  | *Haemophilus pittmaniae* | 0·880 | 0·029 | 0·087 |
|  | *Bacteroides sp CBA7301* | 0·670 | 0·030 | 0·087 |
|  | *Neisseria mucosa* | 1·130 | 0·030 | 0·087 |
|  | *Selenomonas ruminantium* | 0·720 | 0·030 | 0·087 |
|  | *Veillonella nakazawae* | 0·560 | 0·030 | 0·088 |
|  | *Streptococcus urinalis* | 0·630 | 0·030 | 0·088 |
|  | *Mammaliicoccus sciuri* | 0·550 | 0·031 | 0·090 |
|  | *Olsenella sp oral taxon 807* | 1·040 | 0·031 | 0·090 |
|  | *Bacteroides thetaiotaomicron* | 0·530 | 0·032 | 0·090 |
|  | *Porphyromonas crevioricanis* | 0·590 | 0·032 | 0·091 |
|  | *Streptococcus ruminantium* | 0·790 | 0·032 | 0·091 |
|  | *Corynebacterium jeikeium* | 0·650 | 0·033 | 0·092 |
|  | *Propionimicrobium sp Marseille P3275* | 0·680 | 0·033 | 0·092 |
|  | *Gemella haemolysans* | 0·900 | 0·033 | 0·092 |
|  | *Alistipes dispar* | 0·710 | 0·034 | 0·093 |
|  | *Porphyromonas asaccharolytica* | 0·740 | 0·033 | 0·093 |
|  | *Streptococcus salivarius* | 0·780 | 0·034 | 0·093 |
|  | *Porphyromonas cangingivalis* | 0·620 | 0·035 | 0·095 |
|  | *Bacteroides zoogleoformans* | 0·660 | 0·035 | 0·095 |
|  | *Lancefieldella parvula* | 0·670 | 0·035 | 0·095 |
|  | *Selenomonas sp oral taxon 920* | 0·760 | 0·035 | 0·095 |
|  | *Treponema pedis* | 0·690 | 0·035 | 0·096 |
|  | *Clostridium saccharoperbutylacetonicum* | 0·590 | 0·036 | 0·096 |
|  | *Haemophilus influenzae* | 0·830 | 0·036 | 0·097 |
|  | *Paludibacter propionicigenes* | 0·640 | 0·036 | 0·097 |
|  | *Streptococcus oralis* | 0·730 | 0·037 | 0·097 |
|  | *Micrococcus luteus* | 0·680 | 0·037 | 0·098 |
|  | *Neisseria brasiliensis* | 0·880 | 0·037 | 0·098 |
|  | *Corynebacterium vitaeruminis* | 0·670 | 0·038 | 0·099 |
|  | *Bulleidia sp zg 1006* | 0·530 | 0·039 | 0·099 |
|  | *Paraprevotella xylaniphila* | 0·560 | 0·039 | 0·099 |
|  | *Butyrivibrio fibrisolvens* | 0·580 | 0·038 | 0·099 |
|  | *Barnesiella viscericola* | 0·590 | 0·039 | 0·099 |
|  | *Streptococcus constellatus* | 0·630 | 0·039 | 0·099 |
|  | *Streptococcus sp NSJ 72* | 0·640 | 0·039 | 0·099 |
|  | *Bergeyella cardium* | 0·670 | 0·038 | 0·099 |
|  | *Aggregatibacter actinomycetemcomitans* | 0·880 | 0·039 | 0·099 |
|  | *Neisseria bacilliformis* | 0·910 | 0·039 | 0·099 |

Legend to Table 6: Linear contrasts have been constructed and tested for significance using voom:limma approach combined with GMPR normalisation. Adjustment for multiple testing was done using Benjamini-Hochberg false discovery rate within each taxonomic level. Reported are findings with FDR <10% in at least one contrast. MCII = minimum clinically important improvement, RA = rheumatoid arthritis. Note: At baseline and at six weeks MCII- data not shown, no significant differences

**Supplementary Table 7:** Differentially abundant taxa in saliva microbiota between RA patients who did not achieve minimum clinically important improvement (MCII-) at baseline and 12 weeks treatment

| **Taxonomic level** | **Taxa** | **MCII- between baseline and 12 Weeks** | | |
| --- | --- | --- | --- | --- |
|  |  | **logFC** | **P.Value** | **P.adj value** |
| Species | *Actinobaculum sp 313* | 1·270 | 0·005 | 0·090 |
|  | *Actinomyces faecalis* | 1·370 | 0·002 | 0·088 |
|  | *Actinomyces gaoshouyii* | 1·450 | 0·001 | 0·088 |
|  | *Actinomyces howellii* | 1·450 | 0·002 | 0·088 |
|  | *Actinomyces lilanjuaniae* | 1·290 | 0·004 | 0·090 |
|  | *Actinomyces marmotae* | 1·440 | 0·001 | 0·088 |
|  | *Actinomyces procaprae* | 1·300 | 0·004 | 0·090 |
|  | *Actinomyces qiguomingii* | 1·420 | 0·002 | 0·088 |
|  | *Actinomyces radicidentis* | 1·440 | 0·002 | 0·088 |
|  | *Actinomyces respiraculi* | 1·280 | 0·005 | 0·090 |
|  | *Actinomyces slackii* | 1·450 | 0·002 | 0·088 |
|  | *Actinomyces sp 432* | 1·440 | 0·002 | 0·088 |
|  | *Actinomyces sp Chiba101* | 1·560 | 0·001 | 0·088 |
|  | *Actinomyces sp MAS 1* | 1·420 | 0·003 | 0·090 |
|  | *Actinomyces sp Z16* | 1·430 | 0·002 | 0·088 |
|  | *Actinomyces trachealis* | 1·290 | 0·004 | 0·090 |
|  | *Actinomyces weissii* | 1·290 | 0·004 | 0·090 |
|  | *Actinomyces wuliandei* | 1·470 | 0·001 | 0·088 |
|  | *Brachybacterium saurashtrense* | 1·470 | 0·003 | 0·090 |
|  | *Brevibacterium casei* | 1·620 | 0·004 | 0·090 |
|  | *Brevilactibacter coleopterorum* | 1·320 | 0·006 | 0·090 |
|  | *Clostridium sp C1* | 1·610 | 0·003 | 0·090 |
|  | *Cutibacterium avidum* | 1·510 | 0·001 | 0·088 |
|  | *Dialister pneumosintes* | 2·200 | 0·001 | 0·088 |
|  | *Faecalibacterium prausnitzii* | 1·250 | 0·002 | 0·088 |
|  | *Fretibacterium fastidiosum* | 2·190 | 0·005 | 0·090 |
|  | *Fusobacterium sp oral taxon 203* | 1·960 | 0·003 | 0·090 |
|  | *Lancefieldella parvula* | 1·730 | 0·001 | 0·088 |
|  | *Moraxella osloensis* | 2·110 | 0·002 | 0·088 |
|  | *Olsenella uli* | 2·320 | 0·000 | 0·070 |
|  | *Prevotella copri* | 1·170 | 0·006 | 0·099 |
|  | *Propionibacterium freudenreichii* | 1·350 | 0·004 | 0·090 |
|  | *Rothia amarae* | 1·690 | 0·003 | 0·090 |
|  | *Rothia mucilaginosa* | 1·410 | 0·004 | 0·090 |
|  | *Rothia sp ZJ932* | 1·590 | 0·006 | 0·098 |
|  | *Salmonella enterica* | 1·570 | 0·002 | 0·088 |
|  | *Schaalia meyeri* | 1·250 | 0·005 | 0·090 |
|  | *Streptococcus dysgalactiae* | 1·280 | 0·005 | 0·090 |
|  | *Streptococcus pseudoporcinus* | 1·250 | 0·005 | 0·090 |
|  | *Treponema sp OMZ 806* | 2·510 | 0·005 | 0·090 |
|  | *Trueperella pyogenes* | 1·350 | 0·004 | 0·090 |
|  | *Veillonella dispar* | 1·230 | 0·006 | 0·090 |

Legend to Table 7: Linear contrasts have been constructed and tested for significance using voom:limma approach combined with GMPR normalisation. Adjustment for multiple testing was done using Benjamini-Hochberg false discovery rate within each taxonomic level. Reported are findings with FDR <10% in at least one contrast. MCII = minimum clinically important improvement, RA = rheumatoid arthritis. Note: At baseline and at 12 weeks MCII+ data not shown, no significant differences

**Supplementary Table 8:** Differentially abundant stool microbiota between RA patients who achieved minimum clinically important improvement (MCII+) between baseline and 12 weeks treatment

| **Taxonomic level** | **Taxa** | **MCII+ between baseline and 12 weeks** | | |
| --- | --- | --- | --- | --- |
|  |  | **logFC** | **p-value** | **adj. p-value** |
| Phylum | Tenericutes |  |  |  |
|  | Fusobacteria | -0·470 | 0·007 | 0·020 |
| Family | Fusobacteriaceae | -0·410 | 0·007 | 0·020 |
|  | Carnobacteriaceae | -0·790 | 0·000 | 0·005 |
|  | NA.8 | -0·800 | 0·000 | 0·009 |
|  | Pasteurellaceae | -0·990 | 0·000 | 0·009 |
|  | Staphylococcaceae | -0·920 | 0·000 | 0·009 |
|  | Streptococcaceae | -0·610 | 0·000 | 0·012 |
|  | Tissierellaceae | -1·170 | 0·001 | 0·012 |
|  | Mycoplasmataceae | -0·540 | 0·001 | 0·012 |
|  | Leptotrichiaceae | -0·690 | 0·001 | 0·013 |
|  | NA.33 | -0·550 | 0·001 | 0·017 |
|  | Acholeplasmataceae | -1·870 | 0·002 | 0·031 |
|  | NA.2 | -0·490 | 0·002 | 0·033 |
|  | Morganellaceae | -0·540 | 0·005 | 0·059 |
|  | Peptoniphilaceae | -0·440 | 0·006 | 0·067 |
| Genus | Haemophilus | -0·420 | 0·007 | 0·071 |
|  | Streptococcus | -1·330 | 0·000 | 0·017 |
|  | Paraclostridium | -1·150 | 0·001 | 0·017 |
|  | Veillonella | -1·010 | 0·000 | 0·006 |
|  | Granulicatella | -0·990 | 0·001 | 0·019 |
|  | Sarcina | -0·950 | 0·001 | 0·017 |
|  | Paeniclostridium | -0·920 | 0·000 | 0·004 |
|  | Pediococcus | -0·910 | 0·000 | 0·008 |
|  | Lactococcus | -0·850 | 0·000 | 0·004 |
|  | Heyndrickxia | -0·830 | 0·008 | 0·070 |
|  | Gemella | -0·820 | 0·000 | 0·003 |
|  | Fusobacterium | -0·790 | 0·003 | 0·041 |
|  | Schnuerera | -0·770 | 0·000 | 0·003 |
|  | Sedimentibacter | -0·770 | 0·000 | 0·016 |
|  | Mammaliicoccus | -0·770 | 0·000 | 0·014 |
|  | Latilactobacillus | -0·750 | 0·000 | 0·017 |
|  | Pradoshia | -0·700 | 0·010 | 0·082 |
|  | Blattabacterium | -0·700 | 0·001 | 0·020 |
|  | Raoultella | -0·690 | 0·000 | 0·003 |
|  | Buchnera | -0·690 | 0·006 | 0·063 |
|  | Crassaminicella | -0·670 | 0·000 | 0·014 |
|  | Finegoldia | -0·670 | 0·000 | 0·003 |
|  | Lachnospira | -0·650 | 0·002 | 0·034 |
|  | Niallia | -0·650 | 0·012 | 0·093 |
|  | Staphylococcus | -0·640 | 0·001 | 0·021 |
|  | Vagococcus | -0·630 | 0·000 | 0·012 |
|  | Parvimonas | -0·630 | 0·001 | 0·024 |
|  | Carnobacterium | -0·620 | 0·010 | 0·079 |
|  | Sebaldella | -0·590 | 0·003 | 0·045 |
|  | Peptacetobacter | -0·590 | 0·001 | 0·024 |
|  | Polaribacter | -0·570 | 0·002 | 0·036 |
|  | Proteus | -0·570 | 0·001 | 0·019 |
|  | Pasteurella | -0·550 | 0·010 | 0·082 |
|  | Anaerococcus | -0·540 | 0·002 | 0·031 |
|  | Companilactobacillus | -0·530 | 0·004 | 0·047 |
|  | Dolosigranulum | -0·530 | 0·004 | 0·049 |
|  | Lachnoanaerobaculum | -0·530 | 0·005 | 0·055 |
|  | Tissierella | -0·530 | 0·003 | 0·040 |
|  | Tetragenococcus | -0·530 | 0·003 | 0·045 |
|  | Brachyspira | -0·510 | 0·003 | 0·037 |
|  | Caloramator | -0·500 | 0·002 | 0·035 |
|  | Acetoanaerobium | -0·500 | 0·003 | 0·037 |
|  | Oceanobacillus | -0·480 | 0·002 | 0·037 |
|  | Virgibacillus | -0·480 | 0·004 | 0·049 |
|  | Tepidanaerobacter | -0·480 | 0·001 | 0·020 |
|  | Filifactor | -0·470 | 0·004 | 0·047 |
|  | Lysinibacillus | -0·460 | 0·002 | 0·031 |
|  | Halanaerobium | -0·460 | 0·004 | 0·047 |
|  | Hathewaya | -0·450 | 0·002 | 0·037 |
|  | Thermoanaerobacterium | -0·450 | 0·004 | 0·047 |
|  | Ruminiclostridium | -0·450 | 0·002 | 0·034 |
|  | Priestia | -0·440 | 0·005 | 0·055 |
|  | Vallitalea | -0·430 | 0·004 | 0·047 |
|  | Jeotgalibaca | -0·430 | 0·006 | 0·060 |
|  | Leptotrichia | -0·420 | 0·008 | 0·073 |
|  | Serpentinicella | -0·420 | 0·005 | 0·053 |
|  | Ureibacillus | -0·420 | 0·009 | 0·074 |
|  | Pelosinus | -0·420 | 0·008 | 0·070 |
|  | Sutcliffiella | -0·410 | 0·006 | 0·063 |
|  | Caldicellulosiruptor | -0·380 | 0·008 | 0·070 |
|  | Helicobacter | -0·370 | 0·008 | 0·071 |
|  | Oceanidesulfovibrio | -0·350 | 0·007 | 0·065 |
| Species | *Crassaminicella sp. SY095* | 0·360 | 0·009 | 0·078 |
|  | *Fusobacterium nucleatum* | -0·720 | 0·000 | 0·002 |
|  | *Clostridium cochlearium* | -0·960 | 0·000 | 0·002 |
|  | *Bacillus mycoides* | -0·950 | 0·000 | 0·002 |
|  | *Clostridium fermenticellae* | -0·760 | 0·000 | 0·003 |
|  | *Crassaminicella sp. 143 21* | -0·730 | 0·000 | 0·003 |
|  | *Streptococcus mutans* | -0·660 | 0·000 | 0·003 |
|  | *Clostridium sporogenes* | -1·660 | 0·000 | 0·003 |
|  | *Blattabacterium cuenoti* | -0·810 | 0·000 | 0·004 |
|  | *Clostridium saccharoperbutylacetonicum* | -0·640 | 0·000 | 0·006 |
|  | *Sarcina sp. JB2* | -0·790 | 0·000 | 0·006 |
|  | *Clostridium baratii* | -0·860 | 0·000 | 0·006 |
|  | *Clostridium septicum* | -0·810 | 0·000 | 0·006 |
|  | *Clostridium sp. DL VIII* | -0·990 | 0·000 | 0·006 |
|  | *Thermoanaerobacterium sp. RBIITD* | -0·680 | 0·000 | 0·006 |
|  | *Clostridium sp. C5S11* | -0·650 | 0·000 | 0·006 |
|  | *Helcococcus kunzii* | -0·790 | 0·000 | 0·006 |
|  | *Clostridium taeniosporum* | -0·750 | 0·000 | 0·006 |
|  | *Clostridium gasigenes* | -0·770 | 0·000 | 0·006 |
|  | *Vagococcus fluvialis* | -0·800 | 0·000 | 0·006 |
|  | *Granulicatella elegans* | -0·980 | 0·000 | 0·006 |
|  | *Tetragenococcus halophilus* | -0·930 | 0·000 | 0·008 |
|  | *Clostridium sp. JN 1* | -0·640 | 0·000 | 0·008 |
|  | *Clostridium butyricum* | -0·660 | 0·000 | 0·009 |
|  | *Clostridium intestinale* | -0·650 | 0·000 | 0·011 |
|  | *Clostridium sp. CT4* | -0·660 | 0·000 | 0·011 |
|  | *Paraclostridium bifermentans* | -0·590 | 0·000 | 0·011 |
|  | *Veillonella dispar* | -0·890 | 0·000 | 0·011 |
|  | *Clostridium acetobutylicum* | -1·000 | 0·000 | 0·011 |
|  | *Paeniclostridium sordellii* | -0·610 | 0·000 | 0·011 |
|  | *Clostridium kluyveri* | -0·810 | 0·000 | 0·011 |
|  | *Clostridium perfringens* | -0·520 | 0·000 | 0·012 |
|  | *Gottschalkia acidurici* | -0·880 | 0·000 | 0·013 |
|  | *Schnuerera ultunensis* | -0·650 | 0·000 | 0·014 |
|  | *Clostridium chauvoei* | -0·710 | 0·000 | 0·014 |
|  | *Clostridium cellulovorans* | -0·860 | 0·001 | 0·015 |
|  | *Buchnera aphidicola* | -0·560 | 0·001 | 0·016 |
|  | *Clostridium argentinense* | -0·600 | 0·001 | 0·017 |
|  | *Sebaldella termitidis* | -0·600 | 0·001 | 0·017 |
|  | *Sedimentibacter sp. zth1* | -0·550 | 0·001 | 0·017 |
|  | *Mammaliicoccus sciuri* | -0·680 | 0·001 | 0·017 |
|  | *Clostridium pasteurianum* | -0·870 | 0·001 | 0·017 |
|  | *Gracilibacillus sp. SCU50* | -0·500 | 0·001 | 0·019 |
|  | *Lactobacillus iners* | -0·630 | 0·001 | 0·021 |
|  | *Pradoshia sp. D12* | -0·830 | 0·001 | 0·022 |
|  | *Haemophilus parainfluenzae* | -0·640 | 0·001 | 0·023 |
|  | *Caloranaerobacter azorensis* | -1·380 | 0·001 | 0·024 |
|  | *Clostridium sp. C1* | -0·510 | 0·001 | 0·024 |
|  | *Gemella haemolysans* | -0·930 | 0·001 | 0·024 |
|  | *Brachyspira pilosicoli* | -0·930 | 0·001 | 0·024 |
|  | *Clostridium sp. JN 9* | -0·590 | 0·001 | 0·025 |
|  | *Pediococcus acidilactici* | -0·490 | 0·001 | 0·025 |
|  | *Paraliobacillus zengyii* | -0·660 | 0·001 | 0·025 |
|  | *Finegoldia magna* | -0·740 | 0·001 | 0·025 |
|  | *Niallia circulans* | -0·600 | 0·001 | 0·025 |
|  | *Staphylococcus aureus* | -0·570 | 0·001 | 0·025 |
|  | *Clostridium sp. AWRP* | -0·580 | 0·002 | 0·028 |
|  | *Streptococcus gallolyticus* | -0·580 | 0·002 | 0·030 |
|  | *Streptococcus intermedius* | -0·940 | 0·002 | 0·030 |
|  | *Clostridium botulinum* | -0·910 | 0·002 | 0·034 |
|  | *Clostridium carboxidivorans* | -0·510 | 0·002 | 0·035 |
|  | *Clostridium isatidis* | -0·570 | 0·002 | 0·035 |
|  | *Crassaminicella profunda* | -0·670 | 0·002 | 0·035 |
|  | *Fusobacterium ulcerans* | -0·450 | 0·002 | 0·035 |
|  | *Listeria welshimeri* | -0·510 | 0·002 | 0·035 |
|  | *Caloramator sp. E03* | -0·600 | 0·002 | 0·035 |
|  | *Streptococcus sp. oral taxon 061* | -0·460 | 0·002 | 0·036 |
|  | *Thermoanaerobacterium xylanolyticum* | -0·990 | 0·002 | 0·036 |
|  | *Clostridium formicaceticum* | -0·530 | 0·003 | 0·038 |
|  | *Clostridium novyi* | -0·440 | 0·003 | 0·038 |
|  | *Peptoniphilus harei* | -0·540 | 0·003 | 0·038 |
|  | *Veillonella parvula* | -0·570 | 0·003 | 0·038 |
|  | *Clostridium bornimense* | -1·050 | 0·003 | 0·039 |
|  | *Cytobacillus kochii* | -0·480 | 0·003 | 0·040 |
|  | *Halanaerobium praevalens* | -0·610 | 0·003 | 0·041 |
|  | *Staphylococcus haemolyticus* | -0·570 | 0·003 | 0·041 |
|  | *Peribacillus psychrosaccharolyticus* | -0·560 | 0·003 | 0·041 |
|  | *Alkaliphilus sp. B6464* | -0·600 | 0·003 | 0·043 |
|  | *Clostridium tyrobutyricum* | -0·510 | 0·004 | 0·044 |
|  | *Clostridium drakei* | -0·460 | 0·004 | 0·047 |
|  | *Streptococcus thermophilus* | -0·580 | 0·004 | 0·049 |
|  | *Tepidanaerobacter acetatoxydans* | -1·300 | 0·004 | 0·049 |
|  | *Desulfobulbus oralis* | -0·430 | 0·004 | 0·049 |
|  | *Ureibacillus thermosphaericus* | 0·450 | 0·005 | 0·052 |
|  | *Ruminiclostridium herbifermentans* | -0·480 | 0·004 | 0·052 |
|  | *Lactococcus cremoris* | -0·410 | 0·005 | 0·054 |
|  | *Peptacetobacter hiranonis* | -0·710 | 0·005 | 0·056 |
|  | *Vallitalea guaymasensis* | -0·480 | 0·005 | 0·056 |
|  | *Sutterella faecalis* | -0·440 | 0·005 | 0·056 |
|  | *Aeribacillus pallidus* | 0·710 | 0·005 | 0·058 |
|  | *Clostridium aceticum* | -0·490 | 0·006 | 0·060 |
|  | *Desulfovibrio marinus* | -0·440 | 0·006 | 0·060 |
|  | *Streptococcus pseudoporcinus* | 0·400 | 0·006 | 0·060 |
|  | *Anaerococcus prevotii* | -0·780 | 0·006 | 0·060 |
|  | *Gemella morbillorum* | -0·400 | 0·006 | 0·060 |
|  | *Clostridium thermarum* | -0·690 | 0·006 | 0·061 |
|  | *Faecalibacterium prausnitzii* | -0·390 | 0·006 | 0·062 |
|  | *X Ruminococcus gnavus* | 0·670 | 0·006 | 0·062 |
|  | *Filifactor alocis* | -0·660 | 0·006 | 0·062 |
|  | *Acetoanaerobium sticklandii* | -0·380 | 0·006 | 0·062 |
|  | *Bacillus pumilus* | -0·410 | 0·006 | 0·063 |
|  | *Streptococcus oralis* | -0·460 | 0·007 | 0·063 |
|  | *Tissierella sp. JN 28* | -0·830 | 0·007 | 0·063 |
|  | *Hathewaya histolytica* | -0·460 | 0·007 | 0·063 |
|  | *Veillonella atypica* | -0·390 | 0·007 | 0·063 |
|  | *Lachnoanaerobaculum umeaense* | -0·790 | 0·007 | 0·063 |
|  | *Selenomonas sp. oral taxon 920* | -0·460 | 0·008 | 0·072 |
|  | *Bacillus thuringiensis* | 0·450 | 0·008 | 0·073 |
|  | *Desulfolutivibrio sulfoxidireducens* | -0·420 | 0·008 | 0·076 |
|  | *Pseudodesulfovibrio mercurii* | 0·420 | 0·009 | 0·076 |
|  | *Granulicatella adiacens* | 0·390 | 0·009 | 0·076 |
|  | *Erysipelothrix sp. HDW6A* | -0·790 | 0·009 | 0·077 |
|  | *Streptococcus equinus* | -0·570 | 0·009 | 0·078 |
|  | *Streptococcus mitis* | -0·710 | 0·009 | 0·078 |
|  | *Ligilactobacillus salivarius* | -0·840 | 0·009 | 0·078 |
|  | *Metabacillus sediminilitoris* | -0·780 | 0·010 | 0·081 |
|  | *Oceanobacillus zhaokaii* | -0·440 | 0·010 | 0·083 |
|  | *Pasteurella multocida* | -0·500 | 0·010 | 0·083 |
|  | *Clostridium saccharobutylicum* | -0·460 | 0·010 | 0·083 |
|  | *Selenomonas sp. oral taxon 126* | -0·470 | 0·011 | 0·087 |
|  | *Nitrogeniibacter mangrovi* | 0·450 | 0·011 | 0·087 |
|  | *Dolosigranulum pigrum* | 0·440 | 0·011 | 0·091 |
|  | *Enterococcus faecalis* | -0·450 | 0·012 | 0·095 |
|  | *Streptococcus sp. HSISS2* | -1·040 | 0·012 | 0·095 |
|  | *Bacillus weihaiensis* | -1·150 | 0·012 | 0·095 |
|  | *Metabacillus litoralis* | -0·500 | 0·012 | 0·096 |
|  | *Cellulosilyticum sp. WCF 2* | -0·480 | 0·013 | 0·098 |

Legend to Table 8: Linear contrasts have been constructed and tested for significance using voom:limma approach combined with GMPR normalisation. Adjustment for multiple testing was done using Benjamini-Hochberg false discovery rate within each taxonomic level. Reported are findings with FDR <10% in at least one contrast. MCII = minimum clinically important improvement, RA = rheumatoid arthritis. Note: baseline and MCII+ at six weeks not shown, no significant differences

**Supplementary Table 9:** Differentially abundant taxa in stool microbiota between short-term DMARD-treated RA patients (IMRABIOME) at baseline and long-term DMARD-treated RA (NOAR)

| **Taxonomic level** | **Taxa** | **Baseline vs NOAR** | | |
| --- | --- | --- | --- | --- |
|  |  | **logFC** | P.Value. | P.adj Val |
| Species | *Bradyrhizobium sp PSBB068* | -1·526 | 0·000 | 0·000 |
|  | *Enterobacter asburiae* | -0·718 | 0·000 | 0·001 |
|  | *Pseudoprevotella muciniphila* | -0·908 | 0·000 | 0·002 |
|  | *Porphyromonas gingivalis* | -0·723 | 0·000 | 0·002 |
|  | *Prevotella nigrescens* | -0·970 | 0·000 | 0·002 |
|  | *Porphyromonas sp oral taxon 275* | -0·799 | 0·000 | 0·002 |
|  | *Mogibacterium pumilum* | -0·452 | 0·000 | 0·002 |
|  | *Prevotella oris* | -0·959 | 0·000 | 0·004 |
|  | *Butyricimonas virosa* | -1·296 | 0·000 | 0·005 |
|  | *Petrimonas mucosa* | -0·681 | 0·000 | 0·006 |
|  | *Odoribacter splanchnicus* | -1·007 | 0·000 | 0·007 |
|  | *Prevotella denticola* | -0·840 | 0·000 | 0·007 |
|  | *Sodaliphilus pleomorphus* | -0·766 | 0·000 | 0·007 |
|  | *Alistipes indistinctus* | -1·090 | 0·000 | 0·008 |
|  | *Tannerella serpentiformis* | -0·594 | 0·000 | 0·009 |
|  | *Pseudobacter ginsenosidimutans* | -0·531 | 0·000 | 0·011 |
|  | *Parabacteroides goldsteinii* | -0·914 | 0·000 | 0·011 |
|  | *Prevotella buccalis* | -0·908 | 0·000 | 0·011 |
|  | *Slackia heliotrinireducens* | -0·478 | 0·000 | 0·011 |
|  | *Sinorhizobium fredii* | -0·407 | 0·000 | 0·017 |
|  | *Butyricimonas faecalis* | -0·752 | 0·000 | 0·018 |
|  | *Olsenella sp GAM18* | -0·797 | 0·001 | 0·022 |
|  | *Prevotella sp Rep29* | -0·733 | 0·001 | 0·022 |
|  | *Prevotella multiformis* | -0·744 | 0·001 | 0·022 |
|  | *Draconibacterium orientale* | -0·441 | 0·001 | 0·022 |
|  | *Cryptobacterium curtum* | -0·497 | 0·001 | 0·022 |
|  | *Tannerella forsythia* | -0·584 | 0·001 | 0·022 |
|  | *Prevotella ruminicola* | -0·692 | 0·001 | 0·022 |
|  | *Caloranaerobacter azorensis* | -0·418 | 0·001 | 0·025 |
|  | *Prevotella dentalis* | -0·756 | 0·001 | 0·027 |
|  | *Flavonifractor plautii* | 0·501 | 0·001 | 0·032 |
|  | *Enterobacter cloacae* | -0·485 | 0·001 | 0·032 |
|  | *Prevotella intermedia* | -0·674 | 0·001 | 0·037 |
|  | *Rossellomorea marisflavi* | -0·256 | 0·001 | 0·037 |
|  | *Stutzerimonas stutzeri* | -0·313 | 0·001 | 0·040 |
|  | *Prevotella histicola* | -0·589 | 0·002 | 0·043 |
|  | *Aminipila butyrica* | -0·257 | 0·002 | 0·045 |
|  | *Rothia mucilaginosa* | -0·611 | 0·002 | 0·045 |
|  | *Bdellovibrio bacteriovorus* | -0·313 | 0·002 | 0·046 |
|  | *Cloacibacillus porcorum* | -0·526 | 0·002 | 0·046 |
|  | *Rhodothermus marinus* | -0·391 | 0·002 | 0·046 |
|  | *Chitinophaga sp XS-30* | -0·385 | 0·002 | 0·048 |
|  | *Prevotella melaninogenica* | -0·632 | 0·002 | 0·050 |
|  | *Phascolarctobacterium sp Marseille-Q4147* | -0·751 | 0·002 | 0·051 |
|  | *Prevotella sp oral taxon 475* | -0·548 | 0·002 | 0·051 |
|  | *Prevotella enoeca* | -0·623 | 0·003 | 0·053 |
|  | *Porphyromonas asaccharolytica* | -0·721 | 0·003 | 0·055 |
|  | *Denitrobacterium detoxificans* | -0·393 | 0·003 | 0·055 |
|  | *Parolsenella catena* | -0·587 | 0·003 | 0·057 |
|  | *Pseudomonas putida* | -0·304 | 0·003 | 0·057 |
|  | *Selenomonas sputigena* | -0·350 | 0·003 | 0·057 |
|  | *Thermincola potens* | -0·292 | 0·003 | 0·057 |
|  | *Proteiniphilum saccharofermentans* | -0·425 | 0·003 | 0·057 |
|  | *Prevotella herbatica* | -0·644 | 0·003 | 0·057 |
|  | *Alistipes sp dk3624* | -0·832 | 0·003 | 0·057 |
|  | *Geoalkalibacter subterraneus* | -0·307 | 0·003 | 0·057 |
|  | *Buchnera aphidicola* | -0·368 | 0·003 | 0·057 |
|  | *Atopobium sp oral taxon 416* | -0·341 | 0·004 | 0·059 |
|  | *Agrobacterium tumefaciens* | -0·277 | 0·004 | 0·062 |
|  | *Prevotella jejuni* | -0·627 | 0·004 | 0·063 |
|  | *Klebsiella pneumoniae* | -0·900 | 0·004 | 0·064 |
|  | *Olsenella sp oral taxon 807* | -0·411 | 0·004 | 0·064 |
|  | *Paenibacillus albicereus* | -0·339 | 0·004 | 0·066 |
|  | *Bifidobacterium pseudolongum* | -0·427 | 0·005 | 0·067 |
|  | *Coprobacter secundus* | -0·611 | 0·005 | 0·067 |
|  | *Muribaculum intestinale* | -0·541 | 0·005 | 0·067 |
|  | *Alistipes senegalensis* | -0·711 | 0·005 | 0·067 |
|  | *Desulforapulum autotrophicum* | -0·306 | 0·005 | 0·073 |
|  | *Duncaniella dubosii* | -0·498 | 0·005 | 0·073 |
|  | *Paenibacillus cellulosilyticus* | -0·290 | 0·006 | 0·080 |
|  | *Jejubacter calystegiae* | -0·385 | 0·006 | 0·080 |
|  | *Parolsenella massiliensis* | -0·413 | 0·006 | 0·080 |
|  | *Enterobacter hormaechei* | -0·592 | 0·006 | 0·080 |
|  | *Lacticaseibacillus manihotivorans* | -0·315 | 0·006 | 0·080 |
|  | *Rhodocaloribacter litoris* | -0·431 | 0·006 | 0·081 |
|  | *Klebsiella quasipneumoniae* | -0·633 | 0·007 | 0·085 |
|  | *Paludibacter propionicigenes* | -0·376 | 0·007 | 0·088 |
|  | *Paenibacillus kribbensis* | -0·308 | 0·007 | 0·088 |
|  | *Berryella intestinalis* | -0·420 | 0·007 | 0·090 |
|  | *Clostridium sp BNL1100* | -0·227 | 0·008 | 0·090 |
|  | *Lancefieldella parvula* | -0·365 | 0·008 | 0·095 |
|  | *Parafannyhessea umbonata* | -0·355 | 0·008 | 0·095 |
|  | *Stenotrophomonas maltophilia* | -0·260 | 0·008 | 0·095 |
|  | *Aminipila terrae* | -0·234 | 0·008 | 0·095 |
|  | *Citrobacter freundii* | -0·559 | 0·008 | 0·095 |
|  | *Treponema ruminis* | -0·252 | 0·009 | 0·098 |

Legend to Table 9: Linear contrasts have been constructed and tested for significance using voom:limma approach combined with GMPR normalisation. Adjustment for multiple testing was done using Benjamini-Hochberg false discovery rate within each taxonomic level. Reported are findings with FDR <10% in at least one contrast. MCII = minimum clinically important improvement, RA = rheumatoid arthritis

**Supplementary Table 10:** Differentially abundant taxa in stool microbiota between short-term DMARD-treated RA patients (IMRABIOME) at follow-up and long-term DMARD-treated RA (NOAR).

| **Taxonomic level** | **Taxa** | **Follow-up vs NOAR** | | |
| --- | --- | --- | --- | --- |
|  |  | **logFC** | **P.Value** | **adj.P.Val** |
| Species | *Bradyrhizobium sp PSBB068* | -1·249 | 0·000 | 1·38E-06 |
|  | *Enterobacter asburiae* | -0·658 | 0·000 | 0·003 |
|  | *Olsenella sp GAM18* | -0·991 | 0·000 | 0·003 |
|  | *Lancefieldella parvula* | -0·544 | 0·000 | 0·012 |
|  | *Atopobium sp oral taxon 416* | -0·415 | 0·000 | 0·054 |
|  | *Phascolarctobacterium sp Marseille-Q4147* | -0·854 | 0·000 | 0·062 |
|  | *Pseudoprevotella muciniphila* | -0·662 | 0·001 | 0·064 |
|  | *Porphyromonas gingivalis* | -0·521 | 0·001 | 0·064 |
|  | *Slackia heliotrinireducens* | -0·419 | 0·001 | 0·064 |
|  | *Parolsenella catena* | -0·651 | 0·001 | 0·064 |
|  | *Parafannyhessea umbonata* | -0·441 | 0·001 | 0·064 |
|  | *Megasphaera elsdenii* | -0·702 | 0·001 | 0·064 |
|  | *Bifidobacterium eulemuris* | -0·370 | 0·001 | 0·064 |
|  | *Porphyromonas sp oral taxon 275* | -0·567 | 0·001 | 0·076 |
|  | *Butyricimonas virosa* | -0·980 | 0·001 | 0·076 |
|  | *Flavonifractor plautii* | 0·472 | 0·002 | 0·076 |
|  | *Olsenella uli* | -0·409 | 0·001 | 0·076 |
|  | *Selenomonas sp oral taxon 920* | -0·379 | 0·001 | 0·076 |
|  | *Halanaerobium praevalens* | 0·410 | 0·001 | 0·076 |
|  | *Faecalibaculum rodentium* | -0·328 | 0·002 | 0·085 |
|  | *Prevotella nigrescens* | -0·627 | 0·002 | 0·090 |
|  | *Prevotella oris* | -0·678 | 0·002 | 0·090 |
|  | *Pseudomonas putida* | -0·308 | 0·002 | 0·090 |
|  | *Selenomonas sputigena* | -0·354 | 0·002 | 0·090 |
|  | *Olsenella sp oral taxon 807* | -0·431 | 0·002 | 0·090 |
|  | *Rothia mucilaginosa* | -0·574 | 0·003 | 0·093 |
|  | *Paenibacillus cellulosilyticus* | -0·307 | 0·003 | 0·094 |
|  | *Parolsenella massiliensis* | -0·439 | 0·003 | 0·094 |
|  | *Stutzerimonas stutzeri* | -0·285 | 0·003 | 0·095 |
|  | *Mogibacterium pumilum* | -0·298 | 0·003 | 0·097 |
|  | *Aeromonas veronii* | -0·269 | 0·003 | 0·098 |
|  | *Sutcliffiella horikoshii* | 0·241 | 0·003 | 0·098 |

Legend to Table 10: Linear contrasts have been constructed and tested for significance using voom:limma approach combined with GMPR normalisation. Adjustment for multiple testing was done using Benjamini-Hochberg false discovery rate within each taxonomic level. Reported are findings with FDR <10% in at least one contrast. MCII = minimum clinically important improvement, RA = rheumatoid arthritis

**Supplementary Table 11:** Differential abundance of responders vs non-responders in the NOAR cohort

| **Taxa** | **Responders vs non-responders NOAR cohort** | | |
| --- | --- | --- | --- |
|  | **logFC** | **p-value** | **adj. p-value** |
| *Actinomyces sp. Oral taxon 414* | -1·38 | 0·000 | 0·015 |

Legend to Table 11: DAS28-CRP was generated by NOAR participants, it was not scored by a clinician, and therefore serves as a proxy. Responders = improvement DAS28-CRP <2.6, non-responders = improvement DAS28-CRP >2.6. Linear contrasts have been constructed and tested for significance using voom:limma approach combined with GMPR normalisation. Adjustment for multiple testing was done using Benjamini-Hochberg false discovery rate within each taxonomic level. Reported are findings with FDR <10% in at least one contrast.

**Supplementary Table 12:** Increased abundance of Prevotella species in anti-CCP positive patients at baseline compared to anti-CCP negative

| **Taxa** | **p-value** |
| --- | --- |
| *Prevotella melaninogenica* | **0**·**02** |
| *Prevotella histicola* | **0**·**03** |
| *Prevotella sp. Rep29* | **0**·**04** |
| *Prevotella nigrescens* | **0**·**04** |
| *Prevotella fusca* | **0**·**04** |
| *Prevotella dentalis* | **0**·**04** |
| *Prevotella sp. oral taxon 475* | **0**·**04** |
| *Prevotella jejuni* | 0·06 |
| *Prevotella ruminicola* | 0·07 |
| *Prevotella buccalis* | 0·08 |
| *Prevotella sp. WR041* | 0·11 |
| *Prevotella denticola* | 0·11 |
| *Prevotella intermedia* | 0·11 |
| *Prevotella multiformis* | 0·12 |
| *Prevotella copri* | 0·18 |
| *Prevotella enoeca* | 0·39 |

Legend to Table 12: Patient anti-CCP (binary: positive/negative) response compared to prominent *Prevotella* at baseline. Mann-Whitney U test examined anti-CCP positive vs anti-CCP negative patients against the log taxa abundances.

**Confusion Matrices:**

**Supplementary Table 13:** Confusion matrices of stool microbiota, metacyc pathways and KO genes in baseline DMARD-naive patients. a) stool microbiota, b) metacyc pathways, c) kegg orthologs, d) stool microbiota and metacyc pathways, e) stool microbiota and kegg orthologs, and f) comparison of external data stool microbiota from Gupta and colleagues [5]

**Actual**

**Actual**

**Stool Microbiota**

**Stool Metacyc**

**a)**

**b)**

| \|  \| **0** \| **1** \| \| --- \| --- \| --- \| \| **0** \| 11 \| 5 \| \| **1** \| 10 \| 27 \|   **Predicted** | \| **Sensitivity** \| 0·73 \| \| --- \| --- \| \| **Specificity** \| 0·69 \| \| **Precision** \| 0·84 \| \| **Recall** \| 0·73 \|   **Stool KO genes** | \|  \| **0** \| **1** \| \| --- \| --- \| --- \| \| **0** \| 8 \| 13 \| \| **1** \| 11 \| 16 \|   **d)** | \| **Sensitivity** \| 0·59 \| \| --- \| --- \| \| **Specificity** \| 0·38 \| \| **Precision** \| 0·55 \| \| **Recall** \| 0·59 \|   **Microbiota & Metacyc** |
| --- | --- | --- | --- | --- | --- | --- | --- | --- | --- | --- | --- | --- | --- | --- | --- | --- | --- | --- | --- | --- | --- | --- | --- | --- | --- | --- | --- | --- | --- | --- | --- | --- | --- | --- | --- | --- | --- |
| \|  \| **0** \| **1** \| \| --- \| --- \| --- \| \| **0** \| 9 \| 14 \| \| **1** \| 10 \| 15 \|   **c)**  **Predicted** | \| **Sensitivity** \| 0·60 \| \| --- \| --- \| \| **Specificity** \| 0·39 \| \| **Precision** \| 0·52 \| \| **Recall** \| 0·60 \|   **Microbiota & KO genes** | \|  \| **0** \| **1** \| \| --- \| --- \| --- \| \| **0** \| 12 \| 14 \| \| **1** \| 7 \| 15 \|   **f)** | \| **Sensitivity** \| 0·68 \| \| --- \| --- \| \| **Specificity** \| 0·46 \| \| **Precision** \| 0·52 \| \| **Recall** \| 0·68 \|   **Gupta et. al Microbiota** |
| \|  \| **0** \| **1** \| \| --- \| --- \| --- \| \| **0** \| 7 \| 9 \| \| **1** \| 12 \| 20 \|   **e)**  **Predicted** | \| **Sensitivity** \| 0·63 \| \| --- \| --- \| \| **Specificity** \| 0·44 \| \| **Precision** \| 0·69 \| \| **Recall** \| 0·63 \| | \|  \| **0** \| **1** \| \| --- \| --- \| --- \| \| **0** \| 6 \| 2 \| \| **1** \| 14 \| 10 \| | \| **Sensitivity** \| 0·42 \| \| --- \| --- \| \| **Specificity** \| 0·75 \| \| **Precision** \| 0·83 \| \| **Recall** \| 0·42 \| |

**Supplementary Table 14:** Confusion matrices of saliva microbiota, metacyc pathways and KO genes in baseline DMARD-naive patients.: a) stool microbiota, b) metacyc pathways, c) kegg orthologs, d) stool microbiota and metacyc pathways, e) stool microbiota and kegg orthologs

**Actual**

| \|  \| **0** \| **1** \| \| --- \| --- \| --- \| \| **0** \| 13 \| 18 \| \| **1** \| 8 \| 17 \|   **Actual**  **Predicted** | \| **Sensitivity** \| 0·68 \| \| --- \| --- \| \| **Specificity** \| 0·42 \| \| **Precision** \| 0·49 \| \| **Recall** \| 0·68 \|   **Saliva Microbiota**  **Saliva KO genes** | \|  \| **0** \| **1** \| \| --- \| --- \| --- \| \| **0** \| 10 \| 10 \| \| **1** \| 7 \| 21 \|   **a)**  **b)**  **d)**  **c)**  **e)** | \| **Sensitivity** \| 0·75 \| \| --- \| --- \| \| **Specificity** \| 0·50 \| \| **Precision** \| 0·68 \| \| **Recall** \| 0·75 \|   **Saliva Metacyc**  **Microbiota & Metacyc** |
| --- | --- | --- | --- | --- | --- | --- | --- | --- | --- | --- | --- | --- | --- | --- | --- | --- | --- | --- | --- | --- | --- | --- | --- | --- | --- | --- | --- | --- | --- | --- | --- | --- | --- | --- | --- | --- | --- |
| \|  \| **0** \| **1** \| \| --- \| --- \| --- \| \| **0** \| 6 \| 8 \| \| **1** \| 11 \| 23 \|   **Predicted** | \| **Sensitivity** \| 0·68 \| \| --- \| --- \| \| **Specificity** \| 0·43 \| \| **Precision** \| 0·74 \| \| **Recall** \| 0·68 \|   **Microbiota & KO genes** | \|  \| **0** \| **1** \| \| --- \| --- \| --- \| \| **0** \| 7 \| 8 \| \| **1** \| 10 \| 23 \| | \| **Sensitivity** \| 0·70 \| \| --- \| --- \| \| **Specificity** \| 0·47 \| \| **Precision** \| 0·74 \| \| **Recall** \| 0·70 \| |
| \|  \| **0** \| **1** \| \| --- \| --- \| --- \| \| **0** \| 8 \| 8 \| \| **1** \| 9 \| 23 \|   **Predicted** | \| **Sensitivity** \| 0·72 \| \| --- \| --- \| \| **Specificity** \| 0·50 \| \| **Precision** \| 0·74 \| \| **Recall** \| 0·72 \| |  |  |

**Supplementary Table 15:** Confusion matrices of NOAR stool and saliva microbiota, metacyc pathways and KO genes in long-term DMARD-treated patients. a) stool microbiota, b) stool metacyc pathways, c) stool kegg orthologs, d) saliva microbiota, e) saliva metacyc pathways, and f) saliva kegg orthologs.

**Actual**

**Stool Microbiota**

**Stool Metacyc**

**Actual**

| \|  \| **0** \| **1** \| \| --- \| --- \| --- \| \| **0** \| 15 \| 14 \| \| **1** \| 18 \| 15 \|   **Predicted** | \| **Sensitivity** \| 0·45 \| \| --- \| --- \| \| **Specificity** \| 0·52 \| \| **Precision** \| 0·52 \| \| **Recall** \| 0·45 \|   **Stool KO genes** | \|  \| **0** \| **1** \| \| --- \| --- \| --- \| \| **0** \| 12 \| 4 \| \| **1** \| 21 \| 25 \|   **c)**  **b)**  **a)** | \| **Sensitivity** \| 0·54 \| \| --- \| --- \| \| **Specificity** \| 0·75 \| \| **Precision** \| 0·86 \| \| **Recall** \| 0·54 \| |
| --- | --- | --- | --- | --- | --- | --- | --- | --- | --- | --- | --- | --- | --- | --- | --- | --- | --- | --- | --- | --- | --- | --- | --- | --- | --- | --- | --- | --- | --- | --- | --- | --- | --- | --- | --- | --- | --- |
| \|  \| **0** \| **1** \| \| --- \| --- \| --- \| \| **0** \| 18 \| 10 \| \| **1** \| 15 \| 19 \|   **Predicted** | \| **Sensitivity** \| 0·56 \| \| --- \| --- \| \| **Specificity** \| 0·64 \| \| **Precision** \| 0·66 \| \| **Recall** \| 0·56 \| |  |  |
| \|  \| **0** \| **1** \| \| --- \| --- \| --- \| \| **0** \| 11 \| 8 \| \| **1** \| 24 \| 23 \|   **d)**  **Predicted** | \| **Sensitivity** \| 0·49 \| \| --- \| --- \| \| **Specificity** \| 0·58 \| \| **Precision** \| 0·74 \| \| **Recall** \| 0·49 \|   **Saliva Microbiota**  **Saliva KO genes**  **Saliva Metacyc** | \|  \| **0** \| **1** \| \| --- \| --- \| --- \| \| **0** \| 12 \| 9 \| \| **1** \| 23 \| 22 \|   **e)** | \| **Sensitivity** \| 0·49 \| \| --- \| --- \| \| **Specificity** \| 0·57 \| \| **Precision** \| 0·71 \| \| **Recall** \| 0·49 \| |
| \|  \| **0** \| **1** \| \| --- \| --- \| --- \| \| **0** \| 17 \| 11 \| \| **1** \| 18 \| 20 \|   **f)**  **Predicted** | \| **Sensitivity** \| 0·53 \| \| --- \| --- \| \| **Specificity** \| 0·61 \| \| **Precision** \| 0·65 \| \| **Recall** \| 0·53 \| |  |  |
